# Unveiling Alkali‐Induced Redox Modulation: In‐Situ Spectroscopic Insights from RWGS on Alkali‐Modified ZrO_2_‐Supported Cu Catalysts

**DOI:** 10.1002/advs.202506401

**Published:** 2025-10-13

**Authors:** Abdallah I. M. Rabee, Thanh Huyen Vuong, Laura Kraußer, Hayder Abed, Hanan Atia, Nils Rockstroh, Henrik Lund, Stephan Bartling, Evgenii V. Kondratenko, Angelika Brückner, Jabor Rabeah

**Affiliations:** ^1^ Leibniz‐Institut für Katalyse Albert‐Einstein‐Str. 29A 18059 Rostock Germany; ^2^ Chemistry Department Faculty of Science Minia University El‐Minia 61519 Egypt; ^3^ Al Furat Al Awsat Technical University Najaf 54003 Iraq; ^4^ Department Life Light and Matter University of Rostock Albert‐Einstein‐Str. 25 18059 Rostock Germany; ^5^ State Key Laboratory of Low Carbon Catalysis and Carbon Dioxide Utilization Lanzhou Institute of Chemical Physics (LICP) Chinese Academy of Sciences Lanzhou 730000 P. R. China

**Keywords:** Alkali, CO_2_ hydrogenation, Copper, In‐situ spectroscopy, Redox, Reverse water‐gas shift

## Abstract

Alkali‐modified solid catalysts represent a promising class of heterogeneous catalysts for various applications. Despite extensive research, the role of alkali metals remains debated. In this study, Na‐modified ZrO_2_‐supported Cu catalysts are synthesized using different methods for the reverse water‐gas shift (RWGS) reaction to elucidate the role of Na^+^. Catalytic results show that activity strongly depends on the preparation method, with the one‐pot approach yielding three times higher activity than wet impregnation. The one‐pot method significantly enhances Cu^0^ dispersion, preventing the loss of Cu sites due to agglomeration or coverage by Alkali. Although the prevailing view suggests that alkali metals favor the associative mechanism for CO formation. Combined in‐situ studies demonstrate that the redox mechanism is the main pathway for CO formation. In‐situ EPR and in‐situ CO‐DRIFTS results indicate that Na⁺ plays a critical role in facilitating the reoxidation of reduced Cu^0^ sites by CO_2_ dissociation during the reaction, eventually leading to CO formation. The results show that a high density of accessible Cu^0^ sites with enhanced redox activity is essential for achieving superior catalytic performance. These findings are expected to advance the rational engineering of alkali‐modified surfaces, with enriched active sites and finely tuned redox behavior.

## Graphical abstract

Revisiting the prevailing views that alkali only enhances adsorption properties and stabilizes metal particles against sintering, this study highlights the key role of Na^+^ in modulating Cu redox properties. Na^+^ added via wet impregnation (WI) hinders Cu^2+^ reduction but promotes oxidation and leads to Cu site loss by sintering/coverage. In contrast, the one‐pot method retains a high density of accessible Cu sites with enhanced redox properties.

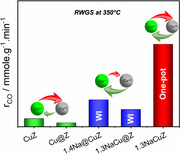

## 1 Introduction

The growing concerns surrounding climate change have driven a strong interest in the development of heterogeneous catalysts for converting CO_2_ into valuable chemicals. Among these, the reverse water gas shift reaction (RWGS) is a promising process that converts CO_2_ into CO, a key feedstock for various chemical syntheses.^[^ ^1^ ^]^ Supported metal nanoparticles (NPs) are highly effective catalysts for this reaction, and their catalytic performance can be further enhanced by modifying them with alkali metals. The impact of alkali metals in heterogeneous catalysis was first reported by Döbereiner in 1845,^[^ ^2^ ^]^ and since then, numerous experimental and theoretical studies have explored their crucial role, especially in hydrogenation reactions such as ammonia synthesis^[^ ^3^ ^]^ and Fischer‐Tropsch synthesis.^[^ ^1^ ^]^ The prevailing theory about the role of alkali metals is that their cations act as electron donors, modifying the local electron density of the active metal.^[^ ^4^, ^5^, ^6^, ^7^, ^8^ ^]^ This modification changes the work function of the active metal, which in turn affects the adsorption energies of intermediates. The increased electron density promotes the back‐donation of electrons from the active metal into the antibonding orbitals of molecules like N_2_ or CO, weakening their bonds and facilitating their dissociative adsorption.^[^ ^9^, ^10^ ^]^ Alternatively, some suggest that alkali ions interact indirectly with active metal sites by surrounding oxygen atoms, stabilizing small metal NPs and atomically dispersed metal sites,^[^ ^11^ ^]^ and preventing sintering in the presence of H_2_.^[^ ^12^, ^13^, ^14^ ^]^ A study has shown that alkali presence leads to a strong Coulombic interaction between alkali cations and H^δ−^ anions, generated from heterolytic H_2_ dissociation.^[^ ^15^ ^]^ This interaction hinders the migration of hydride species, thus preventing the reductive aggregation of metal during hydrogenation at elevated temperatures.^[^ ^15^ ^]^

The role of alkali metals in the RWGS reaction over Cu‐based catalysts has also been explored, with most studies attributing their effects to enhancing CO_2_ adsorption through increased surface basicity^[^ ^16^, ^17^, ^18^, ^19^, ^20^ ^]^ or the formation of oxygen vacancies.^[^ ^21^ ^]^ In a different study, Vogt et al.^[^ ^22^ ^]^ demonstrated that the alkali effect is sensitive to the preparative methods. They observed that in‐situ deposition of KOH on Ni/SiO_2_ improved CO_2_ hydrogenation, while adding the same amount of KOH through wet impregnation led to deactivation. This finding motivated us to explore alternative synthetic approaches for alkali‐modified Cu catalysts, as wet impregnation method is frequently used for adding alkali metals in prior studies.

Additionally, the mechanism of the RWGS reaction over Cu‐based catalysts is still a matter of debate, with two main proposed pathways.^[^ ^17^, ^23^, ^24^, ^25^, ^26^ ^]^ The first is the direct dissociation pathway, or redox mechanism, in which CO_2_ undergoes dissociative adsorption to form surface‐bound CO and O species. The second is the hydrogen‐assisted or associative mechanism, which proceeds via the formation of surface intermediates such as carbonate (CO_3_^2−^), formate (HCOO^−^), and carboxyl/carboxylate (COOH/COO^−^) species. However, the role of alkali in modulating the reaction pathway remains poorly understood. Existing studies suggest a general consensus that alkali addition favors the associative mechanism,^[^ ^25^, ^27^, ^28^, ^29^, ^30^, ^31^, ^32^ ^]^ but its influence on the redox mechanism is still unexplored. These gaps in knowledge highlight the need for further research. Therefore, this study was designed to systematically investigate Na‐modified ZrO_2_‐supported Cu catalysts, aiming to explore alternative synthetic approaches and provide new insights into the role of alkali. To this end, Na‐modified ZrO_2_‐supported Cu catalysts were prepared using different methods, evaluated for the RWGS reaction, and rigorously characterized through both ex situ and in‐situ analytical techniques.

## 2 Results and Discussion

### 2.1 Catalysts Synthesis and Characterization

**Figure 1:**
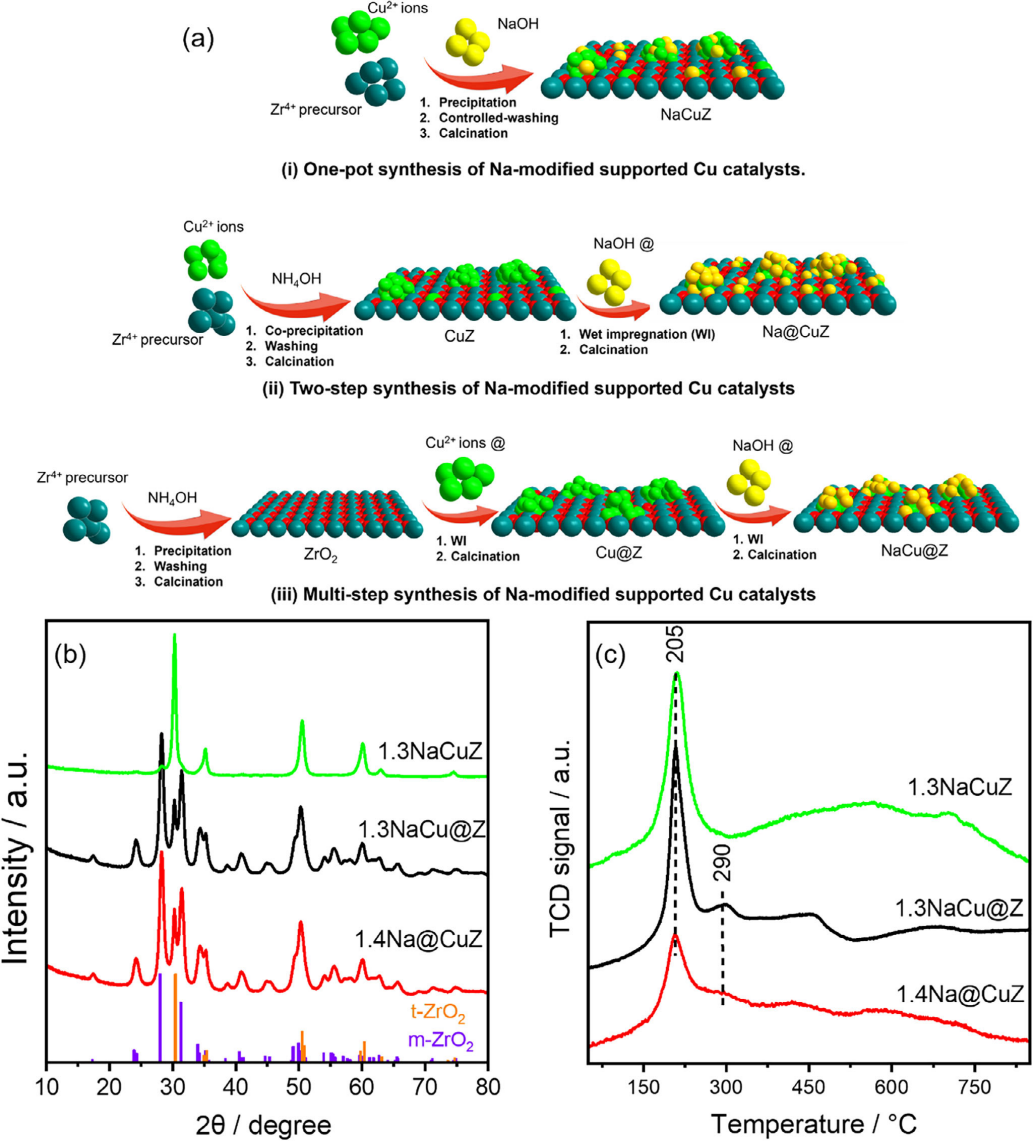
a) Schematic illustration of the synthesis of the different Na‐modified supported Cu catalysts, b) and c) the XRD pattern and H_2_‐TPR profiles, respectively of Na‐modified supported Cu catalysts with similar Na contents.

**Figure** **1**a summarizes the synthesis approaches used for Na‐modified Cu/ZrO_2_ catalysts. Supported Cu was prepared either by co‐precipitation (CuZ) or by wet impregnation (Cu@Z), representing the two most commonly applied methods.^[^ ^33^ ^]^ Subsequent Na addition via wet impregnation facilitated comparison of their structural and catalytic properties with those of the one‐pot synthesis. The actual Cu and Na loadings were listed in Table S1 (Supporting Information). The XRD patterns are shown in Figure S1 (Supporting Information), with Figure 1b comparing catalysts with similar Na^+^ content. All catalysts show both monoclinic (PDF 00‐037‐1484) and tetragonal (PDF 01‐088‐1007) ZrO_2_ phases, and the percentage of each phase is listed in Table S1 (Supporting Information). The results show that adding Cu via co‐precipitation (CuZ, Figure S1a, Supporting Information) or wet impregnation (Cu@Z, Figure S1b, Supporting Information) slightly increases the tetragonal phase compared to bare ZrO_2_ (Figure S1a, Supporting Information). This effect is further enhanced by the addition of Na^+^ (Figure S1a,b, Supporting Information), with the tetragonal phase becoming dominant in the 1.9Na@CuZ (Table S1, Supporting Information). In contrast, all one‐pot synthesized catalysts are dominated by the tetragonal phase (Figure S1c and Table S1, Supporting Information). These findings suggest that Na^+^ addition via the one‐pot method in the presence of Cu^2+^ ions significantly stabilizes the tetragonal phase, even at low Na^+^ content. The stabilization of a high percentage of the tetragonal phase at 0.4 wt.% Na^+^ is not only due to Na^+^, but also to the presence of Cu^2+^. This conclusion is supported by our previous study,^[^ ^34^ ^]^ in which pure ZrO_2_ prepared by the same method and with a similar Na^+^ content (0.36 wt.%) exhibited a predominantly monoclinic phase. Figure S2 (Supporting Information) shows the N_2_ physisorption isotherms, with specific surface area, pore volume, and average pore size summarized in Table S1 (Supporting Information). In catalysts with Na^+^ added via wet impregnation, increasing Na^+^ content reduces surface area and pore volume, suggesting blockage of small pores by Na species. However, the effect on pore size becomes significant only when the Na^+^ content reaches 2 wt.%. Samples prepared by the one‐pot approach also show a decrease in surface area with increasing Na^+^ content, especially at high contents. In contrast, the pore size follows an opposite trend, suggesting that the one‐pot method promotes the formation of structures with larger pores. X‐ray photoelectron spectroscopy (XPS) analysis was used to examine the surface composition and quantify surface Cu and Na contents (Table S2, Supporting Information). The Cu 2p spectra (Figure S3, Supporting Information) show a peak at ≈934 eV, corresponding to Cu^2+^,^[^ ^35^ ^]^ and another at ≈932 eV, from either Cu^+^ or Cu^0^.^[^ ^35^ ^]^ The presence of the Cu^2+^ signal likely results from surface oxidation during air exposure, as the measurements were performed ex‐situ. To distinguish Cu^+^ from Cu^0^, we collected Cu LMM Auger spectra for catalysts prepared by wet impregnation and the one‐pot method with varying Na contents. (Figure S4, Supporting Information). All catalysts exhibit a strong peak at 571.3 eV, characteristic of Cu⁺.^[^ ^35^ ^]^ This peak is slightly broadened with a weak shoulder at 569.4 eV, indicating the presence of some Cu^2+^ species,^[^ ^35^ ^]^ consistent with the Cu 2p spectra. Similar broadening in the one‐pot samples further supports the coexistence of Cu^+^ and Cu^2+^ on the catalyst surface. No peak near 567 eV for metallic Cu^0^ was observed,^[^ ^35^ ^]^ suggesting that Cu^0^ is absent and that the air‐exposed, freshly reduced catalysts are dominated by Cu^+^ and Cu^2+^ species. In the Na 1s region (Figure S5, Supporting Information), all Na‐modified catalysts exhibited a main peak at ≈1071.6 eV (± 0.2 eV), attributed to NaHCO_3_.^[^ ^36^ ^]^ An additional shoulder at ≈1073.6 eV, due to Na_2_O species,^[^ ^37^ ^]^ was observed in the 1.9Na@CuZ, 2NaCuZ, and 1.3NaCu@Z catalysts.

The interaction between Cu and ZrO_2_ was investigated using H_2_‐TPR analysis. Detailed discussions are presented in the supplementary file and Figure S6 (Supporting Information). In general, the addition of Na^+^ shifts the reduction peaks to higher temperatures compared to Na‐free samples. For a better comparison between the synthetic routes, Figure 1c compares the Cu‐containing samples with similar Na^+^ contents. The profiles of 1.4Na@CuZ and 1.3NaCu@Z show two peaks at 205 and 290 °C, indicating a heterogeneous distribution of CuO_x_ species. The high‐temperature reduction peak suggests bulk CuO and/or Cu sites strongly interacting with ZrO_2_ such as Cu incorporated into the ZrO_2_ lattice,^[^ ^38^, ^39^ ^]^ while the lower‐temperature peak suggests highly dispersed small CuO_x_ clusters.^[^ ^38^, ^40^ ^]^ In contrast, the one‐pot synthesized 1.3NaCuZ catalyst demonstrates homogeneous distribution of CuO_x_ species, with a single reduction peak at 205 °C, indicating highly dispersed small CuO_x_ clusters. All profiles showed a broad peak above 350 °C, attributed to the reduction of ZrO_2_ domains.

CO_2_ adsorption was also studied using TPD. Catalysts with similar Na content (Figure S7a, Supporting Information) exhibited similar profiles, indicating that the different preparation methods did not affect CO_2_ adsorption behavior. Since the catalysts have different surface areas, the amount of adsorbed CO_2_ in µmol m^−2^ was calculated and included in the same figure. The values were similar, except for 1.3NaCu@Z, which showed a slightly higher value. To assess the effect of Na content, one‐pot synthesized catalysts with different Na content were examined (Figure S7b, Supporting Information). The profiles remained similar, but the amount of CO_2_ adsorbed increased with Na content, as expected.

**Figure 2:**
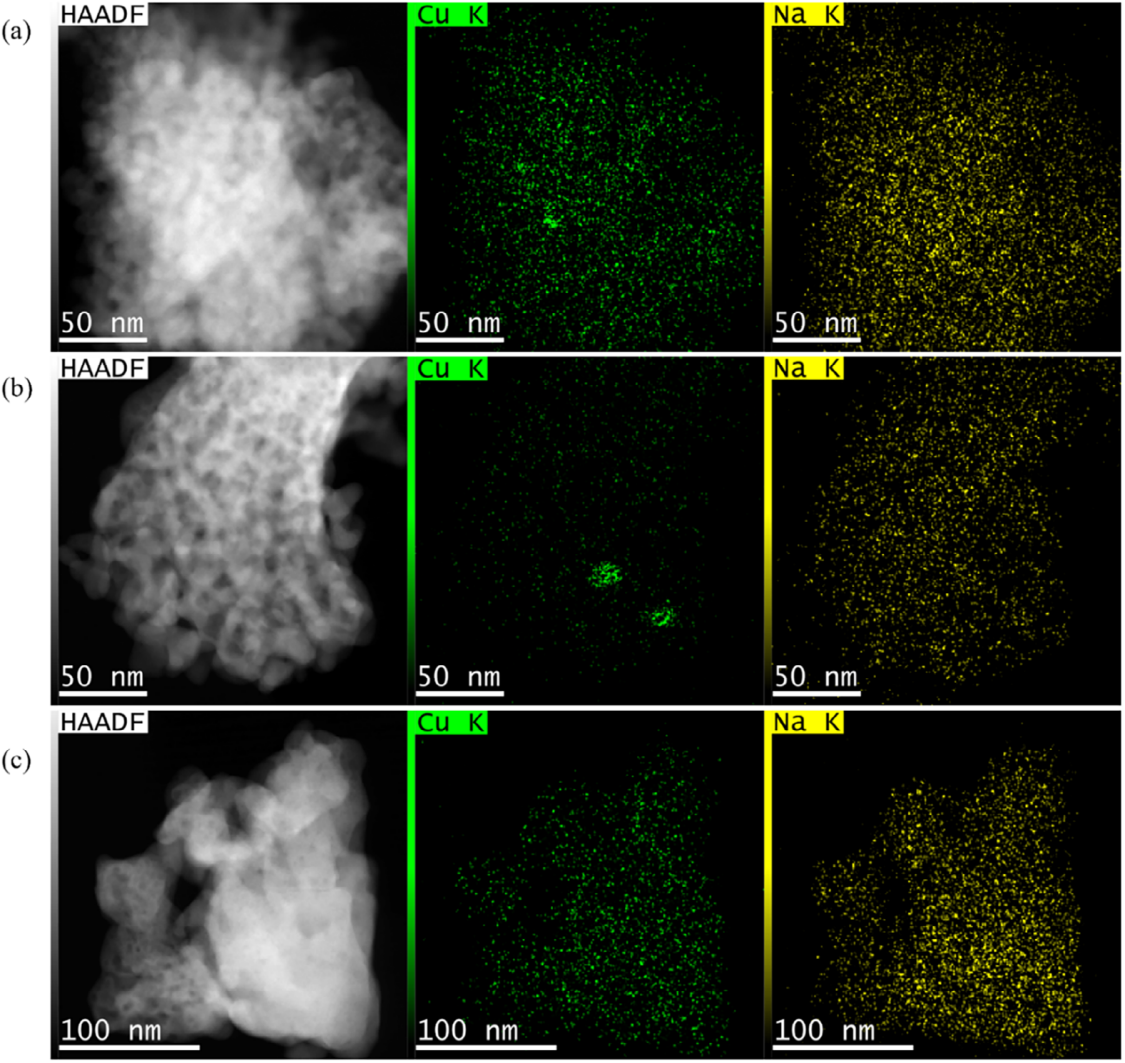
HAADF image and the corresponding EDX elemental mapping images of Cu and Na for a) 1.4Na@CuZ, b) 1.3NaCu@Z, and c) 1.3NaCuZ catalysts, respectively.

The morphology of the catalysts and the distribution of Cu and Na species were further examined using scanning transmission electron microscopy (STEM) imaging and energy dispersive X‐ray (EDX) elemental mapping. Due to the higher atomic number of Zr relative to Cu, it is impossible to detect single Cu atoms or small Cu clusters in high angle annular dark field (HAADF)‐STEM images by contrast. **Figure** **2** presents HAADF micrographs along with the corresponding Cu and Na elemental maps for 1.4Na@CuZ, 1.3NaCu@Z, and 1.3NaCuZ, which have similar Na^+^ content. The EDX elemental maps of Zr and O and their corresponding spectra can be found in the supporting information (Figure S8 and S9–S11, Supporting Information, respectively). Additional representative HAADF images can be found in Figure S12 (Supporting Information). Imaging and EDX analyses reveal a relatively homogeneous distribution of Cu and Na^+^ species in 1.4Na@CuZ and 1.3NaCuZ, suggesting that Cu is predominantly present as highly dispersed CuO_x_ species. However, a slight agglomeration of Cu species is observed on 1.4Na@CuZ, which is far more pronounced on 1.3NaCu@Z, where agglomerations have been observed clearly, consistent with the fact that the wet impregnation method generally produces less dispersed metal species.^[^ ^41^ ^]^ Elemental mapping (Figures S13,S14, Supporting Information) shows that wet‐impregnation of Na^+^ onto CuZ (1.4Na@CuZ) and Cu@Z (1.3NaCu@Z) induces slight CuO_x_ agglomeration. This effect is further supported by the EPR results discussed below.

### 2.2 Catalytic Activity Measurements

**Figure 3:**
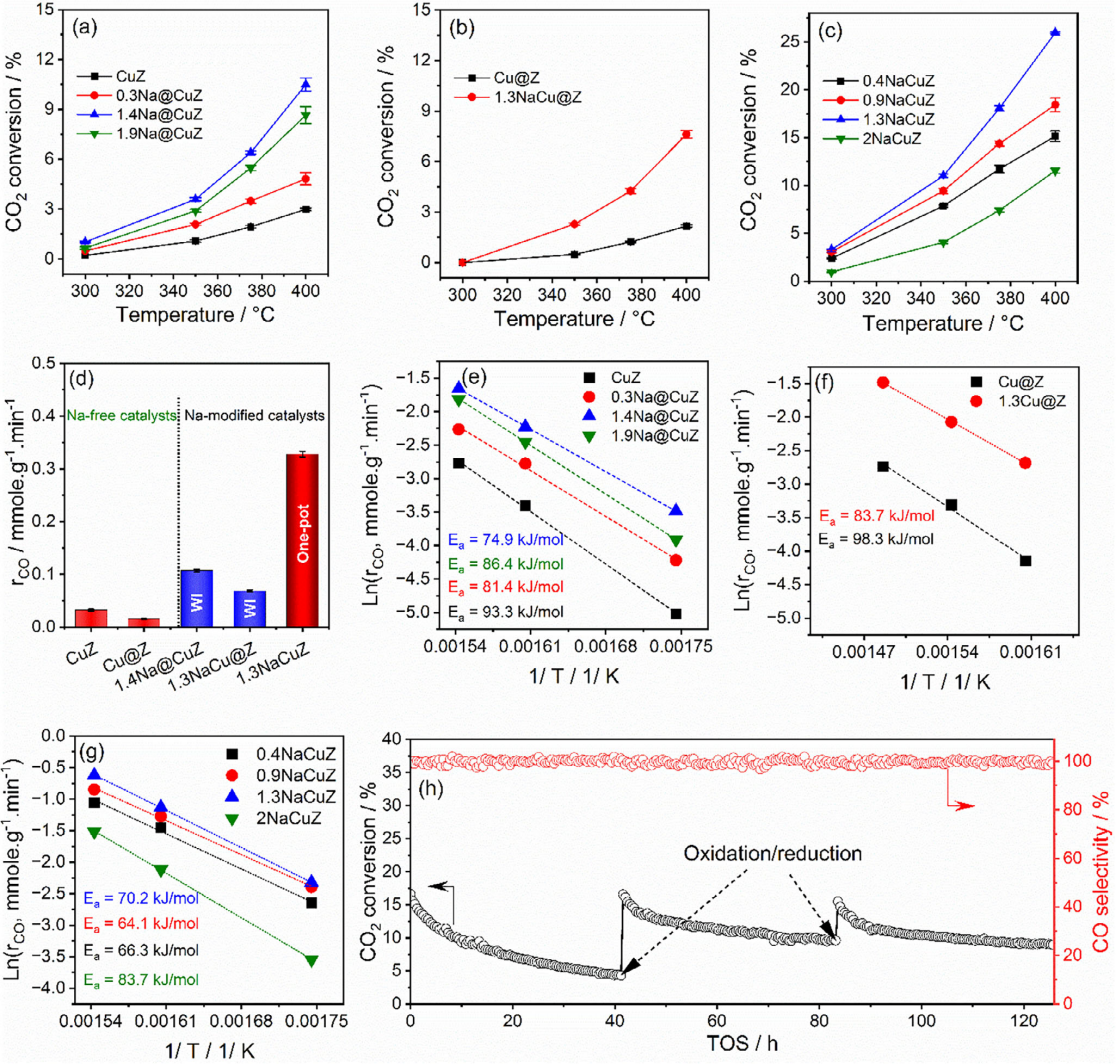
CO_2_ conversion over a) CuZ and xNa@CuZ (Na added via wet impregnation to co‐precipitated CuZ); b) xNaCu@Z (Cu and Na sequentially added to ZrO_2_ via wet impregnation); c) xNaCuZ (one‐pot synthesis with x wt.% Na); d) CO formation rate at 350 °C over Na‐free and Na‐modified catalysts with similar Na content from different synthesis methods, e–g) the Arrhenius plots of CO formation rates, and h) Stability test of 1.3NaCuZ for ≈125 h at 400 °C with a space velocity of 48000 mL·g_cat_^−1^·h^−1^, with re‐activation by oxidation (5% O_2_/N_2_) and reduction (50% H_2_/N_2_) at 20 mL min^−1^ flow for 30 min at the same test temperature. Arrows indicate reactivation points, and error bars represent the standard deviation from four repeated measurements.

The catalytic performance of the bare support and various Na‐free and Na‐modified catalysts in terms of CO_2_ conversion and CO selectivity was evaluated between 300–400 °C with a space velocity of 24000 mL·g_cat_^−1^·h^−1^. The bare support showed no activity (not shown for brevity). **Figure** **3**a compares the CO_2_ conversion of the CuZ catalyst and Na‐modified catalysts. CO_2_ conversion increases with Na contents, reaching a peak at 1.4 wt.%, before slightly declining. The 1.4 wt.%‐Na catalyst showed ≈3.5 times higher conversion than CuZ. A similar trend is observed in Figure 3b for Cu and Na added via wet impregnation (Cu@Z and 1.3NaCu@Z). Interestingly, one‐pot synthesized catalysts (Figure 3c) exhibited higher conversion than those modified via wet impregnation (Figure 3a,b), with 1.3NaCuZ catalyst showing the highest CO_2_ conversion. CO_2_ conversion over the most active 1.3NaCuZ catalyst was also investigated up to 500 °C under the same reaction conditions, and the results are presented in Figure S15 (Supporting Information). The results show that CO_2_ conversion reaches equilibrium at temperatures ≥450 °C. All catalysts demonstrated ~100% CO selectivity throughout the studied range of reaction temperatures. CO formation rates were measured under differential conditions (Figure S16a–c, Supporting Information) with CO_2_ conversion ≤ 11%. For xNa@CuZ and xNaCuZ catalysts, rates were calculated between 300 and 375 °C, while for xNaCu@Z, the range was 350 to 400 °C due to negligible activity at 300 °C. Figure 3d compares CO formation rates at 350 °C, highlighting the superior activity of the one‐pot synthesized 1.3NaCuZ catalyst. Although this study focuses on elucidating the role of Na^+^ in the reaction mechanism rather than optimizing RWGS activity, 1.3NaCuZ remains among the highest‐performing Cu‐based catalysts reported to date (Table S3, Supporting Information). Arrhenius plots (ln rate vs 1/T) in Figure S16d–f (Supporting Information) showed strong linearity, enabling calculation of apparent activation energies (E_a_) and pre‐exponential factors (ln A). For xNa@CuZ and xNaCu@Z (Figure 3e,f), the most active catalysts exhibited the lowest E_a_, consistent with Arrhenius behavior. In contrast, the xNaCuZ series (Figure 3g) showed the highest activity at an intermediate activation energy (E_a_ = 70.2 kJ mol^−1^), rather than the lowest (64.1 kJ mol^−1^), due to a higher pre‐exponential factor (ln A = 12.392 vs 11.064; Figure S16f, Supporting Information). This reflects a compensation effect, where increased ln A offsets the higher E_a_, likely due to greater entropic contributions from improved Cu site accessibility,^[^ ^42^ ^]^ as supported by in‐situ CO‐DRIFTS (vide infra). The linear correlations between ln A and E_a_ observed in both series (Figure S16g,h, Supporting Information) suggest a likely shared rate‐limiting step and reaction mechanism.^[^ ^43^ ^]^

The stability of 1.3NaCuZ was tested at 350 °C (Figure S17, Supporting Information) and 400 °C (Figure 3h) for 125 h. At 400 °C, a higher GHSV of 48000 mL·g_cat_^−1^·h^−1^ was applied because CO_2_ conversion approached equilibrium at the lower GHSV of 24000 mL·g_cat_^−1^·h^−1^. CO selectivity remained near 100% at both temperatures, but CO_2_ conversion declined, with stronger deactivation at 400 °C. Activity was almost fully recovered after oxidation‐reduction at 400 °C but only partially at 350 °C, indicating reversible deactivation requiring high‐temperature oxidation for regeneration. Importantly, the catalyst stability improved after oxidative‐reductive treatments, with less deactivation in subsequent cycles (Figure 3h). TPO analysis (Figure S18, Supporting Information) ruled out coke deposition as the main cause of deactivation. The loss of activity is likely due to surface poisoning by strongly adsorbed carbonaceous species, as later confirmed by in‐situ DRIFTS on Na‐modified catalysts. In addition, sintering‐induced loss of Cu sites may contribute, with subsequent reactivation arising from Cu redispersion. Such redispersion has been reported for Cu‐based catalysts,^[^ ^44^, ^45^, ^46^ ^]^ and is further accelerated by H_2_O even at room temperature.^[^ ^45^ ^]^ Although only O_2_/N_2_ was used here, the high H_2_O detected in the spent catalyst by TPO may have played a similar role. Nonetheless, Cu redispersion in our study remains speculative without direct structural evidence. The stability of 1.4Na@CuZ and 1.3NaCu@Z was also evaluated at 350 °C for 45 h, as shown in Figure S19a,b (Supporting Information), respectively. These catalysts lost 38% and 31% of their initial CO_2_ conversion, respectively, whereas the one‐pot synthesized 1.4NaCuZ lost nearly 50% under the same conditions. In contrast, the Na‐free CuZ catalyst showed only a 21% loss after 45 h (Figure S20, Supporting Information). If deactivation and reactivation are controlled by Cu agglomeration and subsequent redispersion under oxidative conditions, the pronounced activity loss of 1.3NaCuZ can be ascribed to its higher density of accessible Cu sites, as evidenced later by in‐situ CO‐DRIFTS, which renders it more prone to agglomeration than the other Na‐modified catalysts.

### 2.3 In‐Situ Spectroscopic Investigations

#### 2.3.1 In‐Situ EPR Spectroscopic Investigations

**Figure 4:**
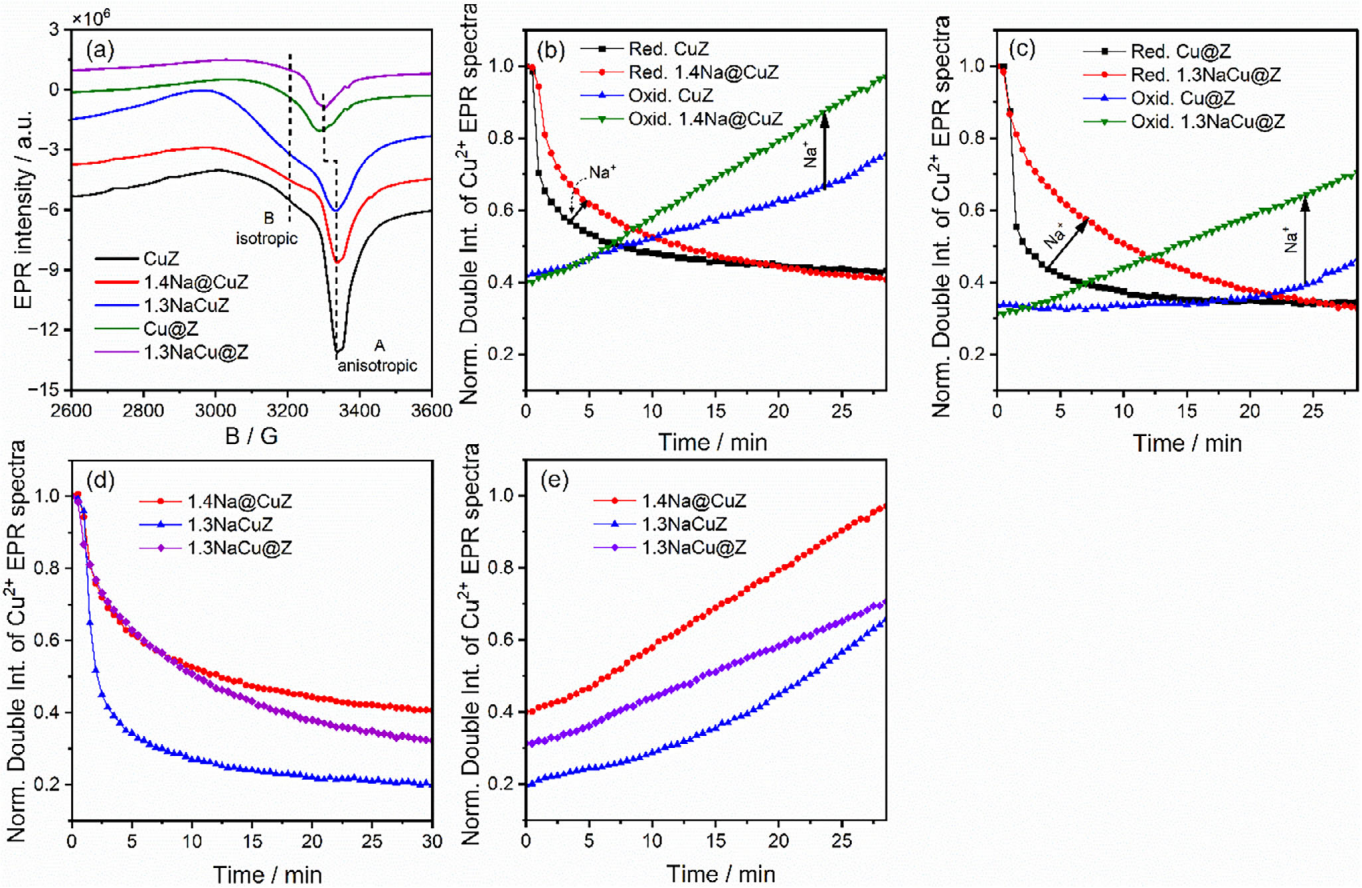
a) In‐situ EPR spectra at −173 °C after preheating in Ar at 300 °C for 1 h. Normalized double integrals of Cu^2+^ EPR spectra recorded at 350 °C under 50% H_2_/Ar flow, followed by 15% CO_2_/Ar flow, for b) CuZ and 1.4Na@CuZ, and c) Cu@Z and 1.3NaCu@Z. Normalized double integrals of Cu^2+^ EPR spectra recorded at 350 °C for Na‐modified catalysts with similar Na^+^ content under d) 50% H_2_/Ar flow and e) 15% CO_2_/Ar flow.

To explain the differences in RWGS catalytic activity, a series of in‐situ EPR investigations were performed, focusing on selected catalysts: Na‐free catalysts (CuZ and Cu@Z) and Na‐modified catalysts (1.4Na@CuZ, 1.3NaCu@Z, and 1.3NaCuZ). To examine the behavior of Cu^2+^ under various gas environments. Initial in‐situ EPR spectra for fresh Na‐free and Na‐modified samples (**Figure** **4**a) revealed that the CuZ sample showed two distinct features: a broad signal (B) ≈3200 G (g‐value = 2.14), attributed to Cu^2+^ ions in small CuO_x_ clusters,^[^ ^47^ ^]^ and another signal (A) ≈3293 G, attributed to Cu^2+^ single sites. After Na^+^ addition (1.4Na@CuZ), the intensity of CuO_x_ signal increased, while Cu^2+^ single sites decreased, indicating slight Cu agglomeration, consistent with STEM‐EDX results (Figure S13, Supporting Information). For the 1.3NaCuZ catalyst prepared via the one‐pot approach, EPR signal broadening was more pronounced, but no Cu agglomeration was observed in EDX (Figure 2a), suggesting interactions between Cu^2+^ sites in small, dispersed clusters. In contrast, when both Cu and Na were added via wet impregnation (Cu@Z and 1.3NaCu@Z), the signals A and B were weaker, indicating the presence of EPR‐silent larger CuO_x_ clusters or NPs, supported by STEM‐EDX results (Figure S14, Supporting Information). These results reveal that, despite similar Cu and Na^+^ contents in these catalysts, differences in the nature of the Cu species were observed, highlighting the significant role of the preparation method in tuning the structure of CuO_x_ sites. The redox behavior was investigated by exposing the catalysts to 50% H_2_/Ar for 30 min, followed by 15% CO_2_/Ar for another 30 min. Figure 4b–e show that upon exposure to H_2_, the normalized double integral of the Cu^2+^ EPR spectra decreased due to the reduction of EPR‐active Cu^2+^ to EPR‐silent Cu^+^ and/or Cu^0^ species. Upon exposure to CO_2_, the intensity increased, indicating that CO_2_ dissociates on the reduced Cu sites, reoxidizing Cu^+^/Cu^0^ back to Cu^2+^. The results also show that Na^+^ addition affects the redox properties differently depending on the method used. For example, Figure 4b,c show that adding Na^+^ via wet impregnation (to form 1.4Na@CuZ and 1.3NaCu@Z) slows the reduction step but enhances reoxidation, particularly for 1.3NaCu@Z. To explore the influence of Na addition method, Figure 4d,e compare the normalized double integral of Cu^2+^ EPR spectra of Na‐modified catalysts with similar Na^+^ content. The detailed spectra are in Figure S21 (Supporting Information). Adding Na^+^ via the one‐pot approach significantly boosts both the rate and degree of reduction (Figure 4d), while maintaining a similar re‐oxidation rate (Figure 4e). In general, regardless for the preparation method, the rate of reduction step by H_2_ was faster than its re‐oxidation by CO_2_. To further assess catalyst stability, we performed RWGS reaction on 1.3NaCuZ for 15 h under the same conditions as the catalytic test, using the in‐situ EPR setup. The spectrum was collected at RT and compared with those of the fresh and freshly reduced samples (Figure S22, Supporting Information). The EPR profile of the spent catalyst closely resembles that of the freshly reduced form, with only a slight increase in the signal intensity of EPR‐active Cu^2+^ species (signal (A)). This suggests partial reoxidation of Cu^0^ to Cu^2+^ during the reaction.

Since EPR is a bulk technique, the spectra in Figure 4 reflect the reduction and oxidation behavior of both surface and bulk/subsurface Cu^2+^ sites. However, only accessible surface Cu sites contribute to the RWGS reaction. Therefore, in‐situ CO‐DRIFTS investigations have been performed (see below).

#### 2.3.2 In‐Situ DRIFTS of CO Adsorption

**Figure 5:**
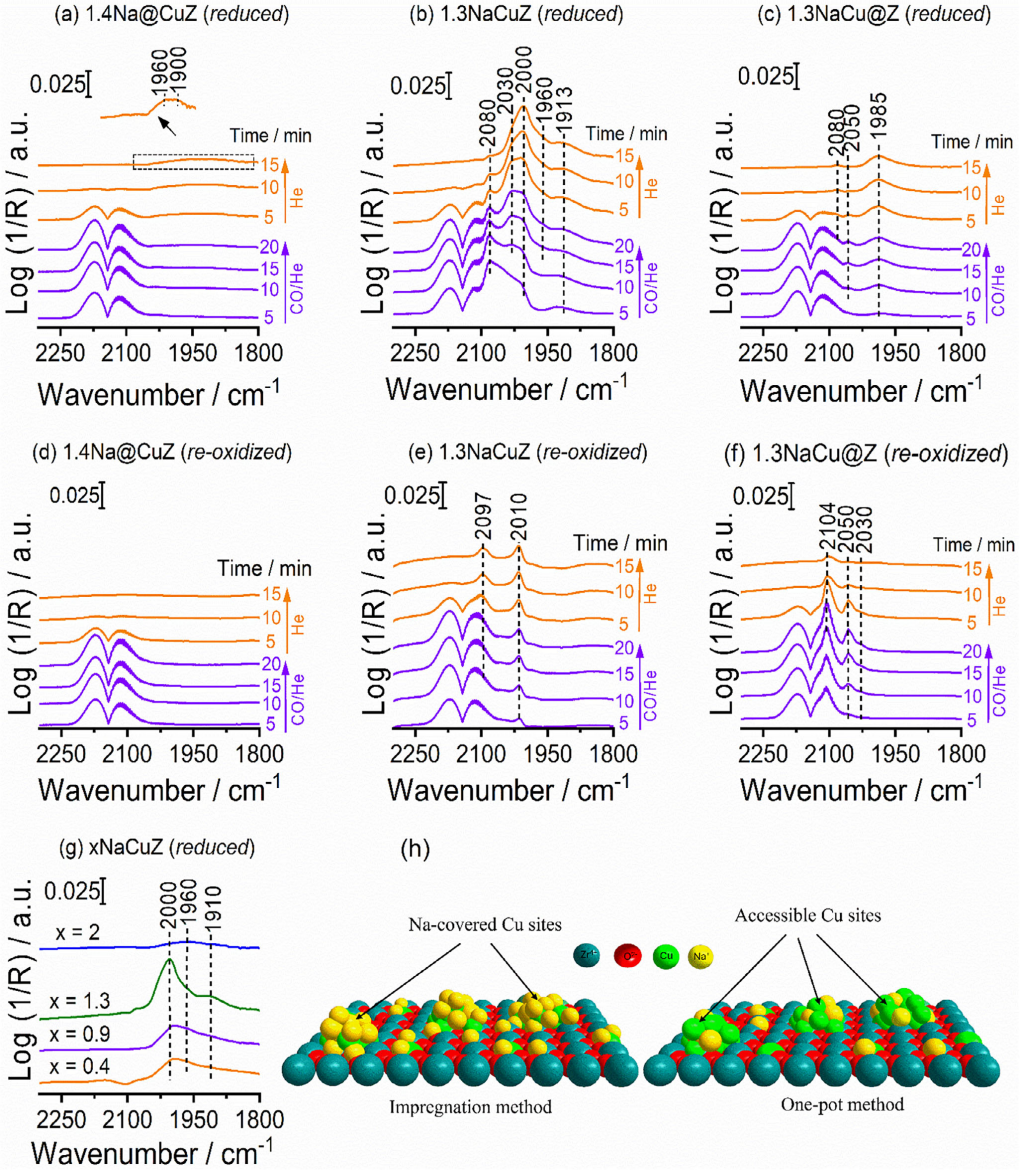
Time‐resolved in‐situ CO‐DRIFTS spectra at 20 °C under a flow of 1% CO/He for 20 min (violet spectra), followed by 100% He for 15 min (orange spectra): a–c) after catalysts were pre‐reduced at 400 °C with 50% H_2_/He for 1 h; d–f) after pre‐treatment at 350 °C with 15% CO_2_/He for 30 min; g) CO adsorption at 20 °C over reduced catalysts from the one‐pot approach (xNaCuZ, where x is Na^+^ content in wt.%); h) Schematic of Cu site accessibility on Na‐modified catalysts prepared by wet impregnation and the one‐pot method.

To probe the surface Cu sites involved in the RWGS reaction, we performed in‐situ CO‐DRIFTS measurements at 20 °C, as CO selectively distinguishes Cu^+^ and Cu^0^, but does not bind to Cu^2+^ at this temperature.^[^ ^48^, ^49^ ^]^ Several experiments on freshly obtained catalysts showed no detectable CO adsorption bands, indicating that Cu is predominantly in the Cu^2+^ state. Representative in‐situ CO‐DRIFT spectra for the Na‐free (CuZ) and Na‐modified (1.3NaCuZ) catalysts are shown in Figure S23 (Supporting Information). Since the oxidation state of Cu under reduced conditions, i.e., just before reaction, is more relevant to catalytic performance than in the fresh state, we then carried out detailed measurements on both H_2_‐reduced catalysts and their CO_2_‐reoxidized forms (treated with CO_2_ at 350 °C for 30 min). The spectra for CO adsorption on Na‐free catalysts are shown in Figure S24 (Supporting Information), while those for catalysts with similar Na^+^ content are presented in **Figure** **5**. When the H_2_‐reduced forms of CuZ and Cu@Z are exposed to CO (violet spectra in Figure S24, Supporting Information), several bands corresponding to CO adsorption appear. These intensities decrease under a He purge (orange spectra) due to the removal of weakly adsorbed CO. The assignments of these bands are summarized in **Table** **1**, with further discussion provided in the Supporting Information. It was observed that the CO adsorption band intensity on the H_2_‐reduced form is slightly higher for Cu@Z (Figure S24b, Supporting Information) than for CuZ, (Figure S24a, Supporting Information), consistent with the higher surface Cu content on Cu@Z (Table S2, Supporting Information). After CO_2_ treatment, the CO band intensity decreases, indicating that CO_2_ reoxidizes reduced Cu to Cu^2+^/Cu^+^ through oxygen from CO_2_ dissociation. This observation aligns with the EPR results and supports the presence of an active redox mechanism. The CO bands on CuZ almost disappear (Figure S24c, Supporting Information), whereas Cu@Z (Figure S24d, Supporting Information) still shows strong CO bands corresponding to Cu^+^ (2110 cm^−1^) and Cu^0^ (2057–2000 cm^−1^). These results indicate that, although CuZ has slightly fewer surface Cu sites, its oxidation behavior by CO_2_ is faster than that of the Cu@Z catalyst. This aligns well with the slightly higher RWGS activity of CuZ compared to Cu@Z (Figure 3d).

**Table 1:** Assignments of the characteristic bands observed in in‐situ CO‐DRIFTS spectra.

wavenumber (cm^−1^)	assignments	refs
2080–2110 cm^−1^	CO@Cu^+^	[48, 50]
2030–2057 cm^−1^	Linearly CO@Cu^0^	[51]
1985–2020 cm^−1^	Bridged CO@Cu^0^ or linearly CO@ negatively charged Cu^0^	[52, 53]
1960–1913 cm^−1^	bridged CO on defective (unsaturated) Cu^0^ sites.	[54, 55]

For catalysts with similar Na content (Figure 5a–c), the CO band intensity is highest for the one‐pot synthesized, H_2_‐reduced 1.3NaCuZ catalyst (Figure 5b). This indicates a high density of accessible surface Cu sites, which also correlates well with its superior catalytic activity. In contrast, the CO band intensities are lower for the 1.4Na@CuZ and 1.3NaCu@Z catalysts. The weak CO band on 1.4Na@CuZ catalyst (Figure 5a) disappears completely after CO_2_ treatment (Figure 5d), suggesting that surface Cu^0^ sites are fully oxidized by CO_2_. On the other hand, the CO_2_‐reoxidized 1.3NaCu@Z catalyst (Figure 5f) still shows distinct CO adsorption bands corresponding to Cu^+^ (2104 cm^−1^) and Cu^0^ (2030–2050 cm^−1^), indicating that the reoxidation of surface Cu sites occurs more slowly on this catalyst. This is also in agreement with the slightly higher activity of 1.4Na@CuZ.

Therefore, the density of accessible Cu sites, as monitored by CO band intensities, and their redox activity, monitored by comparing the CO adsorption band intensities of the same catalyst on its H_2_‐reduced and CO_2_‐reoxidized forms, can only explain the activity trend among the different samples. To further support this hypothesis, CO adsorption on H_2_‐reduced and CO_2_‐reoxidized one‐pot synthesized samples with different Na contents was investigated (Figure S25, Supporting Information). Among these catalysts, 1.3NaCuZ showed the highest CO adsorption band intensity (Table S4, Supporting Information), which correlates well with its superior catalytic activity. On the other hand, the activity difference between 0.4NaCuZ and 0.9NaCuZ cannot be explained by the number of accessible Cu sites, as both show nearly identical CO adsorption band areas on their H_2_‐reduced forms (Table S4, Supporting Information). Instead, the higher activity of 0.9NaCuZ can be attributed to the better redox activity of its surface Cu sites. This is evidenced by the smaller CO band area observed after CO_2_ reoxidation compared to 0.4NaCuZ (Table S4, Supporting Information), indicating that Cu^0^ is more readily reoxidized to Cu^+^ (2090 cm^−1^) and Cu^2+^ (which does not adsorb CO at 20 °C) on 0.9NaCuZ. We also investigated the spent 1.3NaCuZ catalyst after 15 h of RWGS using in‐situ DRIFTS under reaction conditions to assess the stability and oxidation state of surface Cu species. CO adsorption (Figure S26, Supporting Information) showed bands at 2030–1913 cm^−1^ (Cu^0^) and a band at 2090 cm^−1^ (Cu^+^), indicating partial oxidation of Cu^0^ by CO_2_ under RWGS reaction. The reduced intensity of Cu^0^ bands compared with freshly reduced form (Figure 5b) may result from oxidation to Cu^+^ or Cu^2+^, or from partial aggregation. Another point worth mentioning is that Na^+^ addition via wet impregnation reduces the number of accessible Cu sites, as indicated by the lower CO band intensities on 1.4Na@CuZ (Figure 5a) and 1.3NaCu@Z (Figure 5c) compared to their Na‐free counterparts (Figure S24a,b, Supporting Information). This decrease may result from slight CuO_x_ agglomeration following Na addition (Figures S13,S14, Supporting Information) or partial coverage of Cu sites by Na species (Figure 5h).

#### 2.3.3 In‐Situ DRIFTS of CO_2_‐H_2_ Reaction

**Figure 6:**
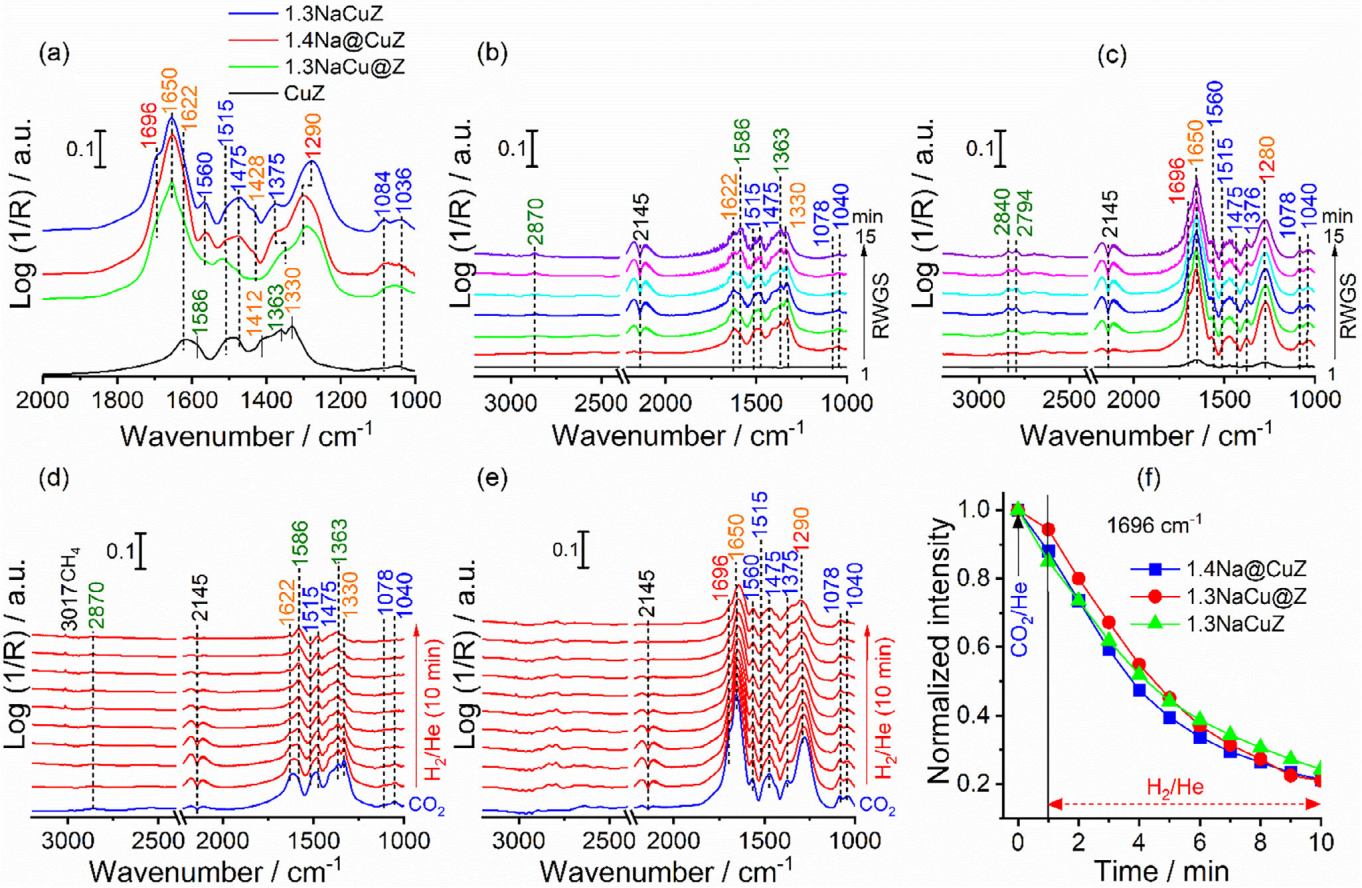
In‐situ DRIFTS spectra: a) after 10 min of CO_2_ adsorption; b,c) during RWGS over reduced CuZ and 1.3NaCuZ catalysts, respectively; d,e) after switching feed gas from 16.6% CO_2_ to 50% H_2_ over reduced CuZ and 1.3NaCuZ, respectively; f) normalized peak intensity of carboxylate species (1696 cm^−1^) during feed gas switching. Condition: p = 1 bar, T = 350 °C, H_2_: CO_2_ = 3:1, total flow rate = 20 mL·min^−1^. The wavenumber values in red, orange, blue, green, and black colors represent the carboxylate, bicarbonates, carbonates, formates, and gaseous CO, respectively.

In‐situ DRIFTS experiments were conducted to investigate surface intermediates and spectator species. For all catalysts studied, steady‐state conditions were reached within a short period (≥10 min). Prolonged DRIFTS experiments for the RWGS reaction over CuZ (Figure S27, Supporting Information) and 1.3NaCuZ (Figure S28, Supporting Information), revealed no additional spectral features after the first 10 min. Therefore, in the following detailed experiments, spectra are presented up to 15 min. First, the adsorption of CO_2_ at 350 °C was studied on selected catalysts (**Figure** **6**a). Over CuZ, the spectra show bidentate bicarbonates (1330, 1412, and 1622 cm^−1^),^[^ ^34^, ^56^ ^]^ monodentate carbonate (1475 and 1508 cm^−1^),^[^ ^34^, ^57^ ^]^ and bidentate formate (1363 and 1586 cm^−1^) species.^[^ ^56^, ^57^, ^58^ ^]^ The formats species may result from CO_2_ interaction with residual H species from the reduction step. The addition of Na^+^, regardless of the preparation method, results in similar spectral features and promotes the formation of new species at 1696 and 1650 cm^−1^, which can be assigned to Na‐stabilized carboxylate and bicarbonate (Na⁺HCO_3_^−^) species, respectively. The formation of ionic bicarbonates due to interactions between CO_2_ with basic OH groups in close proximity to Na⁺. These findings are consistent with Flytzani‐Stephanopoulos et al.,^[^ ^13^, ^59^ ^]^ who reported that alkali addition promotes the formation of OH groups in close proximity.

Figure 6b,c shows the spectra collected under RWGS conditions over CuZ and 1.3NaCuZ, respectively, while spectra collected over 1.4Na@CuZ and 1.3NaCu@Z are shown in Figure S29 (Supporting Information). The spectra over CuZ clearly show the hydrogenation of bicarbonate species (1622 cm^−1^) into formate species (1586 cm^−1^). However, the bicarbonate species at 1650 cm^−1^ appears more stable. The RWGS spectra of catalysts with similar Na^+^ content (Figures 6c and S29) show comparable features with only subtle differences, indicating that CO_2_ adsorption behavior and surface interactions remain unchanged, consistent with the CO_2_‐TPD results (Figure S7, Supporting Information). To examine the effect of Na content, time‐resolved in‐situ DRIFT spectra of one‐pot synthesized catalysts with different Na contents are presented in Figure S30 (Supporting Information). Catalysts with Na^+^ ≥ 0.9 wt.% display similar spectra, dominated by Na‐stabilized bicarbonate and carboxylate species, with minor contributions from formate and carbonate. The 0.4NaCuZ catalyst exhibits all species, but with carbonate bands appearing at a higher intensity. To explore further the role of newly formed species at 1650 and 1696 cm^−1^ in the reaction mechanism, additional experiments were conducted on catalysts with similar Na^+^ content by switching the gas feed from 16.6% CO_2_/He to 50% H_2_/He. To determine whether the decay of adsorbed species was due to hydrogenation to CO or thermal desorption, identical experiments were performed by switching the gas feed from 16.6% CO_2_/He to 100% He. Results for CuZ and 1.3NaCuZ are shown in Figure 6d,e, with detailed comparisons in Figures S31,S32 (Supporting Information). For CuZ (Figure S31, Supporting Information), the intensity of the 1622 cm^−1^ bands decreased under both H_2_ (red) and He (orange) flows, with a more pronounced decay under 50% H_2_/He. The decrease in bicarbonate (1622 cm^−1^) may be due to hydrogenation to formate (1586 and 1363 cm^−1^). For Na‐modified catalysts, the species at 1650 cm^−1^ remain highly stable under both 50% H_2_/He and 100% He, whereas those at 1696 cm^−1^ exhibit decay. However, the similar decay behavior of the 1696 cm^−1^ species across catalysts with similar Na content under either 50% H_2_/He or 100% He (Figure S32, Supporting Information) suggests that this decrease is likely due to thermal desorption. Therefore, these species cannot explain the differences in catalytic activity and thus might be considered spectator species. These highly stable, strongly adsorbed spectator species, which form only after Na addition, could also contribute to the observed higher deactivation of the Na‐modified catalysts (Figure 3h) compared with Na‐free catalysts (e.g., CuZ, Figure S20, Supporting Information).

**Figure 7:**
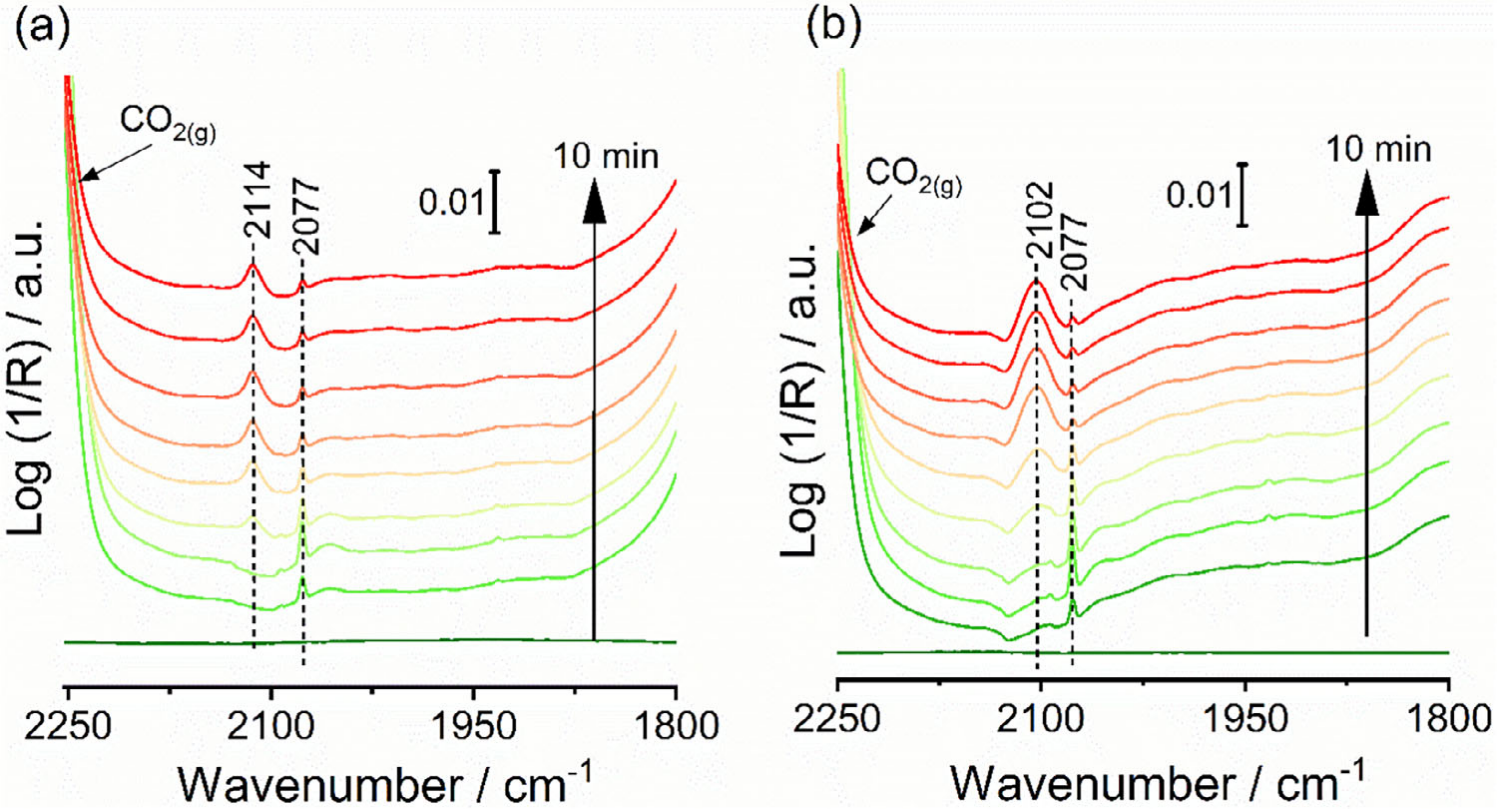
In‐situ DRIFTS spectra collected during the flow of 15% CO_2_/He at 20 °C for 10 min over in‐situ freshly reduced a) CuZ and b) 1.3NaCuZ.

In the switch experiments (Figure 6d,e), CO was detected in the gas phase during CO_2_/He flow (blue spectra) at 350 °C, even after extensive He purging to remove residual H following the reduction step. However, whether the CO forms through a redox process or an associative mechanism involving leftover H species from the reduction step is unclear. Previous studies suggest CO_2_ dissociation can occur spontaneously on pure Cu,^[^ ^60^, ^61^, ^62^ ^]^ even at or below RT,^[^ ^63^, ^64^ ^]^ but others indicate it occurs only on stepped Cu surfaces.^[^ ^61^, ^62^, ^65^ ^]^ To investigate a possible redox mechanism, experiments were designed to study CO_2_ dissociation at 20 °C, preventing CO formation from potential interactions with residual H at higher temperatures. In these experiments, three catalysts were selected: Na‐free CuZ and Na‐modified catalysts with similar Na content (1.3NaCuZ, 1.4Na@CuZ, and 1.3NaCu@Z). The catalysts were reduced at 400 °C for 1 h, cooled to 20 °C, and purged with He for 30 min before introducing a 15% CO_2_/He mixture. The spectra (Figure 7a,b) showed a band at 2077 cm^−1^, which decreased over time while new bands appeared at 2102–2114 cm^−1^. This band is attributed to CO formation via CO_2_ dissociation on Cu^0^ sites. The dissociation process generates CO and O, where the Cu^0^ site involved in the dissociation step becomes oxidized to Cu^+^ by O, and the resulting CO adsorbs on the this newly formed Cu^+^ site. The normalized band areas of these band are shown in Figures S33 (Supporting Information). The lower frequency band on 1.3NaCuZ (2102 cm^−1^ vs 2114 cm^−1^ on CuZ) may result from enhanced back‐donation from Cu⁺ due to Na^+^. These results provide strong evidence for a redox mechanism. On 1.3NaCu@Z and 1.4Na@CuZ, the band at 2077 cm^−1^ (Figure S34, Supporting Information) was weak, and CO adsorption band on Cu^+^ was barely detectable. This is consistent with the low density of accessible Cu^0^ sites on 1.4Na@CuZ, as evidenced by in‐situ DRIFT CO adsorption (Figure 5a). In contrast, although 1.3NaCu@Z exhibits a comparable density of accessible Cu sites to CuZ, based on the in‐situ CO‐DRIFT spectra on their H_2_‐reduced forms (Figures 5c and S24a, Supporting Information), the absence of CO adsorption bands on 1.3NaCu@Z (Figure S34b, Supporting Information) can only be explained by the lower redox activity of its surface Cu sites. This reduced redox behavior, as evidenced by comparing CO adsorption on both its H_2_‐reduced and CO_2_‐reoxidized forms, likely hinders CO_2_ dissociation to occur at 20 °C.

The assignment of the 2077 cm^−1^ band remains debated, with some studies assign it to CO adsorbed on Cu^0^,^[^ ^66^ ^]^ while others attribute it to a combination band of gas‐phase CO_2_.^[^ ^67^, ^68^ ^]^ The absence of this band in CO adsorption experiments (Figure 5) supports the latter hypothesis. However, if it were only due to gas‐phase CO_2_, its intensity should remain constant across all samples, which is not the case. The band is more intense for CuZ and 1.3NaCuZ (Figure 7a,b) compared to others (Figure S34, Supporting Information) during the initial CO_2_ flow but becomes uniform after 10 min, suggesting it might represent physisorbed CO_2_ rather than just gas‐phase CO_2_. This is further supported by the fact that physisorbed CO_2_ is more prone to surface dissociation. The initially high intensity likely results from CO_2_ physically adsorbing on highly defective, energetically exposed Cu^0^ sites, which gradually oxidized into Cu^+^ via the dissociation of physiosorbed CO_2_.

### 2.4 Mechanistic Insights

Studies on alkali free Cu‐based catalysts have shown that both redox and associative pathways (via carboxylate intermediates) contribute to CO_2_ hydrogenation into CO, while the addition of alkali is reported to stabilize the oxygenated intermediates, favoring the associative carboxylate pathway.^[^ ^69^ ^]^ Similarly, studies on Cu‐ and Fe‐based catalysts show that alkali addition shifts the reaction mechanism from a redox to an associative pathway, with formate species proposed as key intermediates.^[^ ^17^, ^18^, ^19^, ^20^ ^]^ Alkali has been shown to promote both the formation and decomposition of formate species.^[^ ^19^, ^20^ ^]^

Our study shows the formation of Na‐stabilized carboxylate species (1696 cm^−1^ band), but its similar intensity and reactivity across all catalysts with similar Na content (Figure 6f) suggest that it does not explain the differences in catalytic activity (Figure 3d). Additionally, negligible formate formation rules out the formate pathway. Since RWGS is the reverse of the WGS reaction, insights from WGS catalysts are valuable. Flytzani‐Stephanopoulos et al.^[^ ^70^ ^]^ found that alkali increases the density of active ‐OH groups, significantly enhancing reactivity. Similarly, our study shows a strong bicarbonate species band at 1650 cm^−1^ (Figure 6a), indicating a higher ‐OH group density. However, these bicarbonate species are highly stable and do not explain the observed increase in catalytic activity.

In‐situ EPR results indicate that Cu^2+^ can be reversibly oxidized and reduced by CO_2_ and H_2_, respectively, suggesting the redox mechanism may be active in both alkali‐free and alkali‐modified catalysts. The results show that Na⁺ enhances the re‐oxidation of Cu^+^/Cu^0^ to Cu^2+^ by CO_2_ compared to Na‐free catalysts (Figure 4b,c). In general, the reoxidation steps were slower than the reduction steps for all the studied catalysts, suggesting that CO_2_ dissociation and the subsequent reoxidation of reduced sites are rate‐determining in the redox mechanism. In‐situ DRIFTS CO adsorption studies on both H_2_‐reduced and CO_2_‐reoxidized catalysts provide robust evidence for a redox mechanism. Detailed analysis of these results reveals that catalytic performance is closely linked to the density and redox activity of accessible reduced surface Cu sites. The in‐situ DRIFTS study of CO_2_ adsorption on freshly reduced catalysts at 20 °C (Figure 7a,b) provides direct experimental evidence of CO_2_ dissociation on surface Cu^0^ into CO and O, demonstrated by the detection of CO adsorption bands on Cu^+^. This further supports the idea that the redox mechanism is the main reaction pathway. Therefore, we propose that metallic Cu^0^, formed during the reduction step, serves as the main active site for RWGS. Due to the relatively fast reduction kinetics compared to reoxidation by CO_2_ (Figure 4d–e), Cu^0^ remains the predominant surface species under RWGS conditions. This conclusion is further supported by in‐situ EPR and DRIFTS studies of the spent catalysts, which show evidence of partial reoxidation to Cu^+^/Cu^2+^ but confirm that most Cu remains in the metallic state during the reaction. The dissociation of CO_2_ on Cu^0^ is initiated by electron transfer from metallic Cu^0^ to the π orbitals of the CO_2_ molecule, which weakens the C═O bonds and induces molecular bending.^[^ ^71^ ^]^ This bending is crucial for activating CO_2_ toward dissociation into CO and O.

Comparing the catalytic activity of catalysts with similar Na content highlights the important role of the preparation method in modulating performance. Na^+^ addition via wet impregnation limits direct CO_2_ dissociation through the redox pathway, as it decreases the availability of Cu^0^ sites due to agglomeration and/or surface coverage. In contrast, the one‐pot synthesis method yields a higher density of accessible Cu^0^ sites with enhanced redox behavior, promoting more effective CO_2_ activation via the redox mechanism. The combination of the highest density of accessible surface Cu sites and their efficient redox properties, as demonstrated by in‐situ DRIFTS CO adsorption studies, is likely responsible for the superior activity of these one‐pot synthesized catalysts.

## 3 Conclusion

In this study, we investigated the synthesis of Na‐modified ZrO_2_‐supported Cu catalysts using different preparation methods. Na‐free catalysts were prepared via co‐precipitation and wet impregnation, while Na‐modified catalysts were synthesized by adding Na through two approaches: (i) wet impregnation and (ii) a one‐pot method, where NaOH served as both the precipitating agent and Na source. Catalysts prepared by the one‐pot method showed a catalytic activity in CO_2_ conversion approximately three times higher than those prepared by wet impregnation. In‐situ EPR identified highly dispersed CuO_x_ clusters, except for the sample prepared by wet impregnation, where Cu NPs were formed (as confirmed by STEM‐EDX). The absence of a clear correlation between catalytic activity and CO_2_ adsorption behavior, whether measured by CO_2_‐TPD or by in‐situ DRIFTS under reaction conditions, weakens the support for proposing an associative mechanism for CO production. In‐situ EPR analysis revealed that Na^+^ played a key role in tuning the redox behavior of the catalysts. Adding Na via wet impregnation decreased the reduction rate while enhancing the re‐oxidation step through CO_2_ dissociation. In contrast, the one‐pot method enhanced both the rate and extent of reduction, while maintaining similar re‐oxidation behavior to wet impregnation. Detailed in‐situ DRIFTS CO adsorption studies on H_2_‐reduced and CO_2_‐reoxidized catalysts showed that Na⁺ added via wet impregnation tends to decrease the number of accessible Cu sites due to agglomeration or surface coverage. However, it enhances the re‐oxidation of the remaining accessible Cu^0^ sites by CO_2_ dissociation (CO_2_ → CO + O), explaining the overall positive effect of Na addition. In contrast, adding Na^+^ through the one‐pot method significantly improves both the accessibility and redox activity of the active Cu sites. Combined results from in‐situ EPR and DRIFTS studies suggest that the redox mechanism is the main pathway on both Na‐free and Na‐modified catalysts.

## Conflict of Interest

The authors declare no conflict of interest.

## Supporting information



Supporting Information

## Data Availability

The data that support the findings of this study are available from the corresponding author upon reasonable request.
