# Supplementary material for: Unveiling Alkali‐Induced Redox Modulation: In‐Situ Spectroscopic Insights from RWGS on Alkali‐Modified ZrO2‐Supported Cu Catalysts
Source: Adv Sci (Weinh). 2025 Oct 13;13(2):e06401. doi: 10.1002/advs.202506401 (PMC12786349; doi:10.1002/advs.202506401)
Supplement: Supplementary file 1 — Supporting Information [file ADVS-13-e06401-s001.pdf]

Supporting Information

Unveiling Alkali-Induced Redox Modulation: In-Situ Spectroscopic Insights from RWGS on Alkali-Modified ZrO_2-Supported Cu Catalysts

Abdallah I. M. Rabee,[1,2] Thanh Huyen Vuong,[1] Laura Kraußer,[1] Hayder Abed,[1,3] Hanan Atia,[1] Nils Rockstroh,[1] Henrik Lund,[1] Stephan Bartling,[1] Evgenii V. Kondratenko,[1] Angelika Brückner,[1,4] Jabor Rabeah [1,5*]*

[1] Leibniz-Institut für Katalyse, Albert-Einstein-Str. 29A, 18059 Rostock, Germany

[2] Chemistry Department, Faculty of Science, Minia University, El-Minia, 61519, Egypt

[3] Al Furat Al Awsat Technical University, Najaf 54003, Iraq

[4] Department Life, Light and Matter, University of Rostock, Albert-Einstein-Str. 25, 18059, Rostock, Germany

[5] State Key Laboratory of Low Carbon Catalysis and Carbon Dioxide Utilization, Lanzhou Institute of Chemical Physics (LICP), Chinese Academy of Sciences, Lanzhou, 730000, P. R. China

1. Materials and Methods

1.1. Chemicals

Zirconium (IV) oxychloride octahydrate ($ZrOCl_2.8H_2O$, 99.9%, Alfa Aesar)), copper nitrate hexahydrate ($Cu(NO_3)_2 \cdot 6H_2O$, >99.0%, Sigma-Aldrich), ammonia solution (28-30%, Sigma-Aldrich), and sodium hydroxide solution (>99.1%, Fisher Chemical,) were used for the preparation of catalysts. All chemicals were used without further treatment.

1.2. Catalyst preparation

Na-modified $CuZrO_2$ catalysts were prepared using two different methods. Initially, bare ZrO_2 and $CuZrO_x$ with a nominal copper loading of 1 wt.% were synthesized via co-precipitation with NH_4OH. For the preparation of $CuZrO_x$, 64.7 g of $ZrOCl_2 \cdot 8H_2O$ and 0.91 g of $Cu(NO_3)_2 \cdot 6H_2O$ were dissolved in 500 ml of distilled water, followed by the dropwise addition of a 1 M NH_4OH solution until the pH reached 9 at RT. For bare ZrO_2 (5 g), the same procedure was used, except the addition of $Cu(NO_3)_2 \cdot 6H_2O$. In both cases, the resulting precipitates were allowed to stand in contact with the mother liquor for 24 h at room temperature, then filtered, washed with distilled water, and dried at 100°C for 12 h. The dried material was ground and calcined at 600°C (with a heating rate of 2°C/min) for 3 h under synthetic air flow. Subsequently, Na-modified $CuZrO_2$ catalysts with nominal Na loadings ranging from 0.5 to 2 wt.% were prepared using the first approach, in which Na^+ was added via wet impregnation. Typically, 4.0 g of $CuZrO_2$ powder were added to 40 mL of aqueous NaOH solutions with concentrations of 21, 61, and 87 mM to achieve Na loadings of 0.5, 1.4, and 2 wt.%, respectively. The mixture was heated at 70°C in a water bath until complete evaporation. This series of catalysts is hereafter referred to as xNa@CuZ, where 'x' denotes the actual Na loading, and 'Cu' and 'Z' in CuZ represent Cu and ZrO_2, respectively. The '@' symbol indicates that Na is added to CuZ via the wet impregnation method. An additional sample with nominal Na and Cu loadings of 1 wt% was prepared using the previously synthesized bare ZrO_2. In this sample, Cu was added first, followed by Na, using the wet impregnation method, with a calcination step at 600°C for 2 hours between the additions. This catalyst is designated as xNaCu@Z, where the presence of 'Na' and 'Cu' before the '@' symbol indicates that both were added to the support via wet impregnation.

In the second method, NaOH served both as a precipitating agent and the Na^+ source for the one-pot synthesis of Na-modified $CuZrO_2$ catalysts. The procedure for $CuZrO_2$ preparation was the

same as before, except that NaOH was used instead of NH_4OH, with 1 M NaOH added dropwise during the precipitation until the pH reached 12. After precipitation, a systematic and well-controlled washing process was applied to adjust the sodium content of the final catalysts. The Na^+ content was varied based on the number and duration of washing cycles, with four cycles employed in this study. In each cycle, 600 ml of distilled water were used. The precipitate was then dried at 100°C for 12 h, ground, and calcined at 600°C (2°C/min) for 3 h under synthetic air flow. These Na-modified $CuZrO_2$ catalysts, prepared by the one-pot method, are designated as xNaCuZ, where "x" indicates the actual Na^+ loading (wt.%). The absence of the symbol '@' in the designation indicates that all constituents of the catalyst were prepared in a one-pot synthesis.

1.3. Catalyst characterization and in-situ investigations

1.3.1. Catalyst characterization.

Prior to characterization, the catalysts were reduced at 400 °C in a 50 vol% H_2/N_2 atmosphere for 2h with a total flow rate of 30 mL·min, and afterwards, immediately transferred to glove box for storage in Ar atmosphere.

1.3.1.1. Elemental analysis

The inductively coupled plasma optical emission spectroscopy (ICP-OES) method was employed to determine the bulk elemental composition of Cu and Na. The measurements were conducted with a 715-ES ICP emission spectrometer (Varian, Palo Alto, CA, USA). Before analysis, the samples were dissolved in a mixture of HF and aqua regia at 200 °C and 60 bar through microwave-assisted digestion.

1.3.1.2. N_2 adsorption-desorption analysis

Brunauer-Emmett-Teller (BET) surface area, pore volume, and average pore diameter measurements were carried out by N_2 adsorption at 77 K using a Micromeritics ASAP 2010 instrument. Typically, 100 mg of sample were placed in the analysis tube and degassed at 200 °C for 4 h prior to exposure to N_2 gas.

1.3.1.3. Powder X-ray diffraction (PXRD)

PXRD characterizations of all samples were carried out on a Panalytical X'Pert PRO diffractometer equipped with a X'Celerator RTMS detector using Ni-filtered Cu-Kα radiation (λ = 0.154 nm) operating at 40 kV and 40 mA. Data was collected stepwise (0.021°/s) in the range of 10- 80º (2 theta) with a divergence slit of 2°. Peak positions and profiles were fitted with Pseudo-Voigt functions using the HighScore Plus software package (Panalytical). Phase identification was done using the PDF-2 database of the International Center of Diffraction Data (ICDD). For quantitative analysis, Rietveld refinement was performed in HighScore plus. Single crystal structures of the identified compounds were obtained from the ICSD database (ICSD entries: 18190 monoclinic ZrO2, 23928 tetragonal zirconia). Their structures were fitted against the obtained powder diffraction data by refinement of scaling factors, lattice parameters, Caglioti peak shape parameters (W, U, V), background (6th-polynomial) and zero-point error, respectively.

1.3.1.4. X-ray Photoelectron Spectroscopy (XPS)

The XPS (X-ray Photoelectron Spectroscopy) measurements were performed on an ESCALAB 220iXL (Thermo Fisher Scientific) with monochromated Al Kα radiation (E = 1486.6 eV). Samples are prepared on a stainless-steel holder with conductive double-sided adhesive carbon tape. The measurements are performed with charge compensation using a flood electron system combining low energy electrons and Ar^+ ions ($p_{Ar} = 1\times10^{-7}$ mbar). The electron binding energies are referenced to the C 1s core level of carbon at 284.8 eV (C-C and C-H bonds). For quantitative analysis, the peaks were deconvoluted with Gaussian-Lorentzian curves using the software Unifit 2023. The peak areas were normalized by the transmission function of the spectrometer and the element specific sensitivity factor of Scofield.

1.3.1.5. H_2-Temperature programmed reduction (TPR)

H_2-TPR measurements were conducted using a Micromeritics Autochem II 2920 instrument. In a typical experiment, 100 mg of fresh catalyst (unreduced) was pretreated in a flow of synthetic air (50 mL·min^{-1}) at 400 °C for 30 min. The sample was then flushed under Ar for 30 min and cooled to room temperature (RT). After reaching RT, the catalyst was exposed to a 10% H_2/Ar flow (50 mL·min^{-1}) while the temperature was ramped up to 900 °C at a rate of 10 K/min. H_2 consumption was monitored using a thermal conductivity detector (TCD). Calibration of the TCD through TPR of CuO facilitated the quantitative evaluation of H_2 consumption.

1.3.1.6. CO_2-Temperature programmed desorption (TPD)

CO_2-TPD experiments were performed using a Micromeritics 3Flex instrument. Nearly 80 mg of catalyst was loaded into a U-shaped quartz reactor and pretreated by heating from RT to 300 °C at a rate of 20 °C/min under a He flow (50 mL/min). The temperature was maintained at 300 °C for 30 min to remove any adsorbed water. The sample was then cooled to 100 °C in the same He flow. At 100 °C, the sample was exposed to a gas mixture containing 1.2% CO_2 in He with a total flow rate of 50 mL/min for 90 min to for CO_2 adsorption. To remove physisorbed CO_2, the sample was subsequently flushed with pure He (50 mL/min) for 60 min at 100 °C. Desorption was carried out by ramping the temperature from 100 °C to 600 °C at a rate of 10 °C/min under He flow (50 mL/min). The temperature was then held at 600 °C for 30 min. The effluent gases were analyzed using a calibrated thermal conductivity detector (TCD).

1.3.1.7. Temperature programmed oxidation (TPO)

For the TPO experiment, the measurement was done using 3Flex instrument connected with an online Quadrupol mass spectrometer (Balzers Omnistar). 50 mg of the spent catalysts were loaded in U shaped quartz reactor. The sample was heated from room temperature to 700°C in the flow of 5 % O_2/He (50 ml/min) with a temperature ramp rate of 10 K/min and the temperature was constant at 700 ~C for 30 min. The signals were continuously recorded via Quadrupol mass spectrometer.

1.3.1.8. Scanning Transmission electron microscopy (STEM)

STEM measurements were carried out utilizing a probe aberration-corrected JEM-ARM200F (JEOL, Corrector: CEOS) operating at 200 kV. The microscope is equipped with a JED-2300 (JEOL) energy-dispersive x-ray spectrometer (EDX) having a silicon drift detector (dry SD60GV). EDX elemental maps are depicted after applying net count fitting using the software Analysis Station (JEOL). For STEM imaging, both High-Angle Annular Dark Field (HAADF) and Annular Bright Field (ABF) detectors were employed. The solid samples were deposited onto a holey carbon-supported Ni grid (mesh 300) without any pre-treatment and subsequently transferred to the microscope for analysis.

1.3.2. In-situ investigations

1.3.2.1. In-situ Electron paramagnetic resonance (EPR) measurements

EPR measurements were performed by an X-band cw-spectrometer ELEXSYS 500-10/12 (Bruker) with a microwave power of 6.3 mW, a modulation frequency of 100 kHz, and modulation amplitude up to 5 G. In-situ EPR experiments were carried out in a home-made quartz plug-flow reactor (3.0 mm inner diameter and 0.5 mm wall thickness). The reactor was connected to a gas-dosing device with mass flow controllers (Bronkhorst) at the inlet and a quadrupole mass spectrometer (Omnistar, Pfeiffer Vacuum GmbH) at the outlet for online product analysis. For each in-situ EPR experiment, 50 mg of catalyst mixed with 50 mg of quartz and a total feed gas flow of 25 mL.min^{-1} were used. Initially, the catalyst was reduced in flowing 50% H_2/Ar at 300 °C for 30 min, followed by flushing with Ar at 25 °C and spectra were recorded at -173 °C. Then, the pre-reduced catalysts were exposed to the following sequence: 1) heating at 300 °C in 15% CO_2 for 30 min, cooling to 25 °C under continuous flow of CO_2, flushing at 25 °C with 25 mL.min^{-1} of Ar for 15 min, and recording spectra at -173 °C; 2) re-reduction at 300 °C in pure H_2 for 15 min, exposure to a flow of CO_2 + H_2 in Ar (CO_2/H_2 ratio = 1/3) at 300 °C for 30 min, cooling to 25 °C under continuous flow of CO_2 + H_2, then flushing with 25 mL.min^{-1} of Ar for 15 min at 25 °C, and recording spectra at -173 °C.

1.3.2.2. In-situ diffuse reflectance infrared Fourier transform spectroscopy (DRIFTS)

In-situ DRIFTS were collected on a Nicolet 6700 FTIR spectrometer using a high-temperature Praying Mantis reaction cell (Harrick) with CaF_2 windows, equipped with a temperature control unit (Eurotherm). Each spectrum was obtained by averaging 64 scans recorded with a resolution of 4 cm^{-1}. Typically, ca. 50 mg of the as-synthesized catalyst powder were deposited on a layer of 70 mg SiC. First, the cell was flushed with He (13 mL.min^{-1}) while the temperature was increased to 400 °C at a rate of 10 K.min^{-1}. Subsequently, the catalyst surface was reduced for 120 min under a flow of 50% mixture H_2 in He (13 mL.min^{-1}) at 400 °C. Thereafter, the cell was flushed with He (13 mL·min^{-1}) at 400 °C. The reaction was performed in a temperature range between 350 and 400 °C with a mixture of H_2:CO_2:He = 3:1:2 at a total flow rate of 20 mL·min^{-1}. The background spectra were taken under He flow at each reaction temperature. The intensity of the signals is given in log(1/R) scale, where $R = I_r/I_b$ represents the ratio between the single beam spectra of the sample at reaction conditions and the single beam spectra of the sample before reaction at a specific temperature.

A series of in-situ DRIFTS experiments was conducted to investigate the behavior of the adsorbed species to alternating CO_2 and H_2 environments at 400 °C. First, the catalyst was reduced as previously described. After reduction, it was purged thoroughly with He flow at 400 °C for 4 hours to remove any adsorbed H_2. Once the purging was complete, the catalyst was exposed to a flow of 16.6 vol% CO_2 in He at a flow rate of 18 $mL.min^{-1}$ for 10 min. After this, the gas was switched to 50% H_2 in He (also at 18 mL·min), and spectra were collected every minute to monitor the interaction between the catalyst and the alternating gas environments.

For the in-situ DRIFTS experiments involving CO adsorption, the studies were conducted on fresh, reduced, and re-oxidized (by CO_2 flow) forms of the catalysts. For the CO adsorption on the fresh catalyst, the sample was first purged with He while being heated to 400 °C and maintained at this temperature for 1 hour. It was then cooled to room temperature (20 °C) under He flow at a rate of 13 $mL.min^{-1}$. A background spectrum was recorded at this point. Next, a gas mixture of 1% CO in He was introduced into the sample cell at a flow rate of 13 $mL.min^{-1}$ for 30 minutes. During this time, spectra were collected every 5 min for a total of 20 min. After CO exposure, the sample was purged with 100% He at the same flow rate (13 $mL.min^{-1}$) for 15 min, during which spectra were collected every 5 min to monitor the flushing of gaseous CO and assess the adsorption strength of the CO species. For the CO adsorption experiments over H_2-reduced samples, the catalysts were first reduced as previously described, followed by the same procedure used for CO adsorption on the fresh samples. For CO adsorption on re-oxidized samples, the catalysts were first reduced, then oxidized by flowing CO_2 at 350 °C for 30 min, before following the same procedure used for the fresh samples. In both cases, each CO-DRIFT spectrum was recorded at 20 °C.

1.4. Catalyst evaluation

The performance of the catalysts was assessed in a quartz fixed-bed reactor at atmospheric pressure over a temperature range of 300 to 400 °C. Typically, 50 mg of the sample with a particle size of ca. 250-315 μm diluted with 450 mg of quartz were placed inside the reactor between two layers of 500 mg quartz. Reduction of catalysts was performed at 400 °C in 50 vol% H_2/N_2 with a flow rate of 20 $mL.min^{-1}$. After cooling to reaction temperature, a $CO_2/H_2/N_2$ = 1/3/2.7 mixture was fed to the reactor with total flow rate of 20 $mL.min^{-1}$. The flow rate was controlled using mass-flow controllers (Bronkhorst). Each gas had a purity of more than 99.99%. The effluent gases were

analyzed using an online gas chromatograph (Shimadzu 2014) equipped with Molsieve 5 Å (Agilent), PoraPLOT Q (Agilent), TCD, FID, and a methanizer. CO and CO_2 were converted into CH_4 through the methanizer and analyzed using the FID detector. The conversion of CO_2 was calculated from the inlet and outlet molar flow rates, as shown in equation (1). The selectivity to CO was calculated using equation (2). The rates of CO formation (r_{CO}) normalized by the total catalyst mass and Cu mass were calculated by equation (3) and (4), respectively.

$$X_{CO2} = \frac{F_{CO2,in} - F_{CO2,out}}{F_{CO2,in}} \times 100\% \quad (1)$$

$$S_x = \frac{F_{x,out}}{F_{CO2,in} - F_{CO2,out}} \times 100\% \quad (2)$$

$$r_{CO} = \frac{X_{CO2} \times S_{CO} \times F_{CO2}}{10000 \times m_{cat.}} \quad (3)$$

$$r_{CO} = \frac{X_{CO2} \times S_{CO} \times F_{CO2}}{10000 \times m_{Cu}} \quad (4)$$

where X_{CO2} is the CO_2 conversion (%), S_x is the selectivity to x product (%), where x is CO or CH_4, and F is a molar flow rate of CO_2 or product (x).

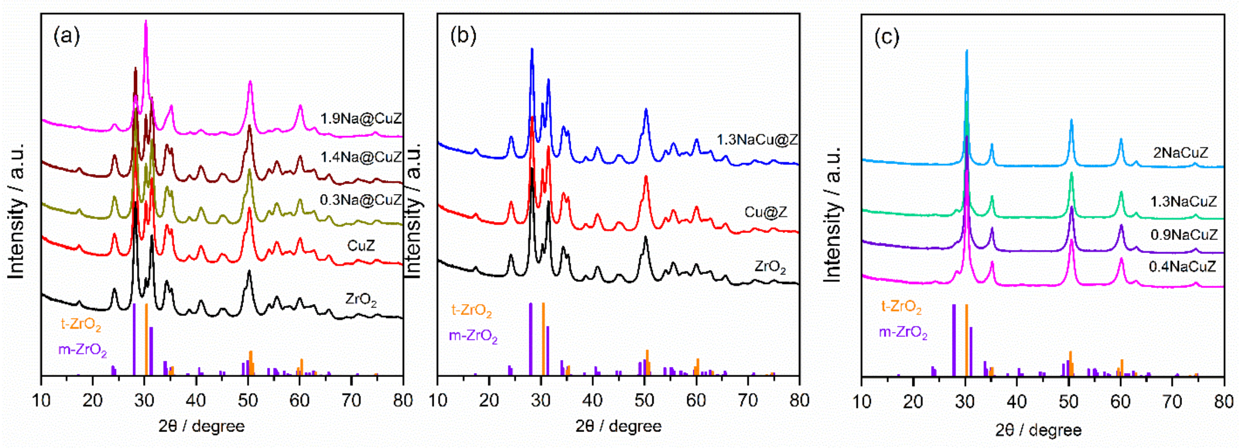

Figure S1. XRD patterns of (a) pure ZrO_2, CuZ, and xNa@CuZ samples (where x represents wt% Na). In these samples, CuZ was prepared via co-precipitation, and Na was added by wet impregnation; (b) xNaCu@Z samples (where x represents wt% Na) in which both Cu and Na were sequentially added via wet impregnation (the diffraction pattern of pure ZrO_2 is included for comparison); and (c) xNaCuZ samples (where x represents wt% Na) prepared using the one-pot synthesis approach.

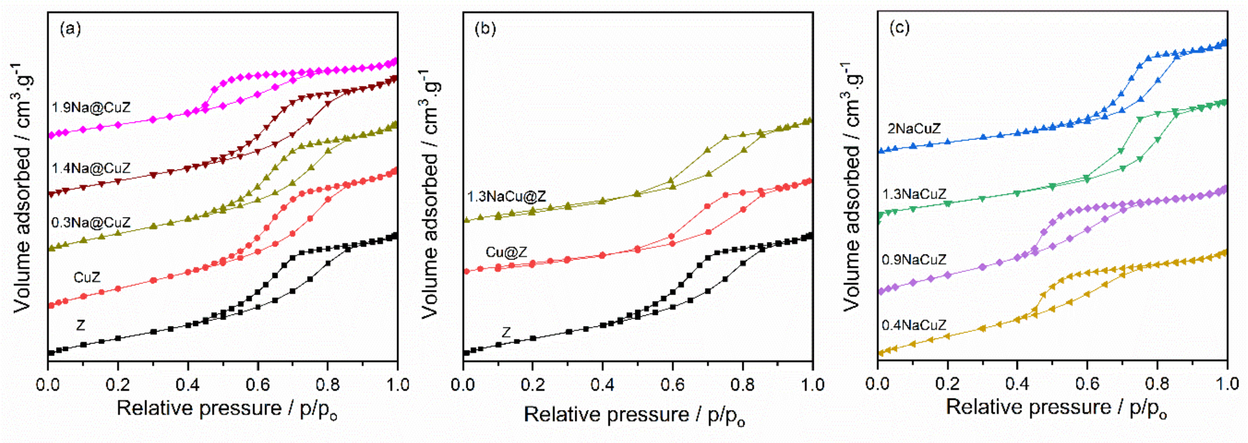

Figure S2. N_2 adsorption-desorption isotherms of (a) pure ZrO_2, CuZ, and xNa@CuZ samples (where x represents wt% Na). In these samples, CuZ was prepared via co-precipitation, and Na was added by wet impregnation; (b) xNaCu@Z samples (where x represents wt% Na) in which both Cu and Na were sequentially added via wet impregnation (the isotherm of pure ZrO_2 is

included for comparison); and (c) xNaCuZ samples (where x represents wt% Na) prepared using the one-pot synthesis method.

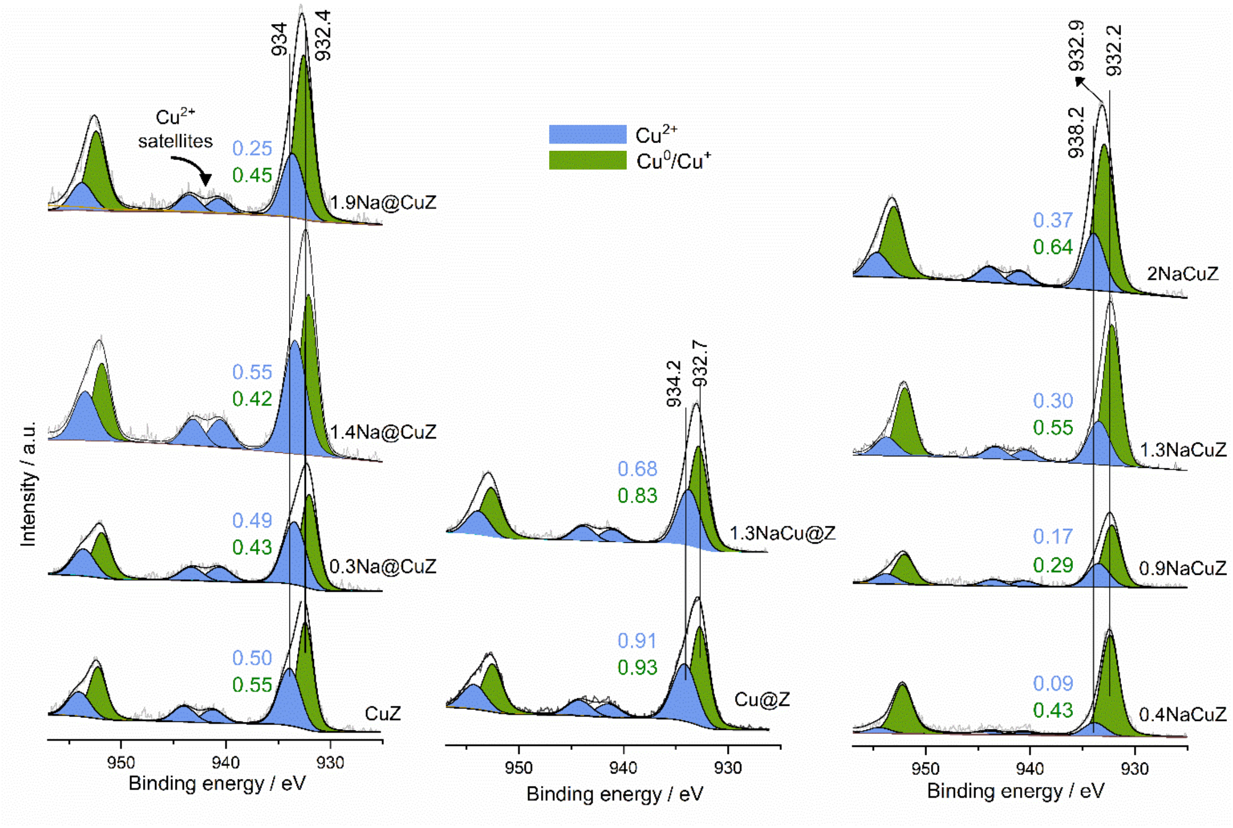

Figure S3. High-resolution XPS spectra of the Cu 2p region for freshly reduced Na-modified catalysts prepared via different synthetic routes. The blue and green numbers indicate the relative percentages (%) of Cu^{2+} and $Cu^{+/0}$ species, respectively.

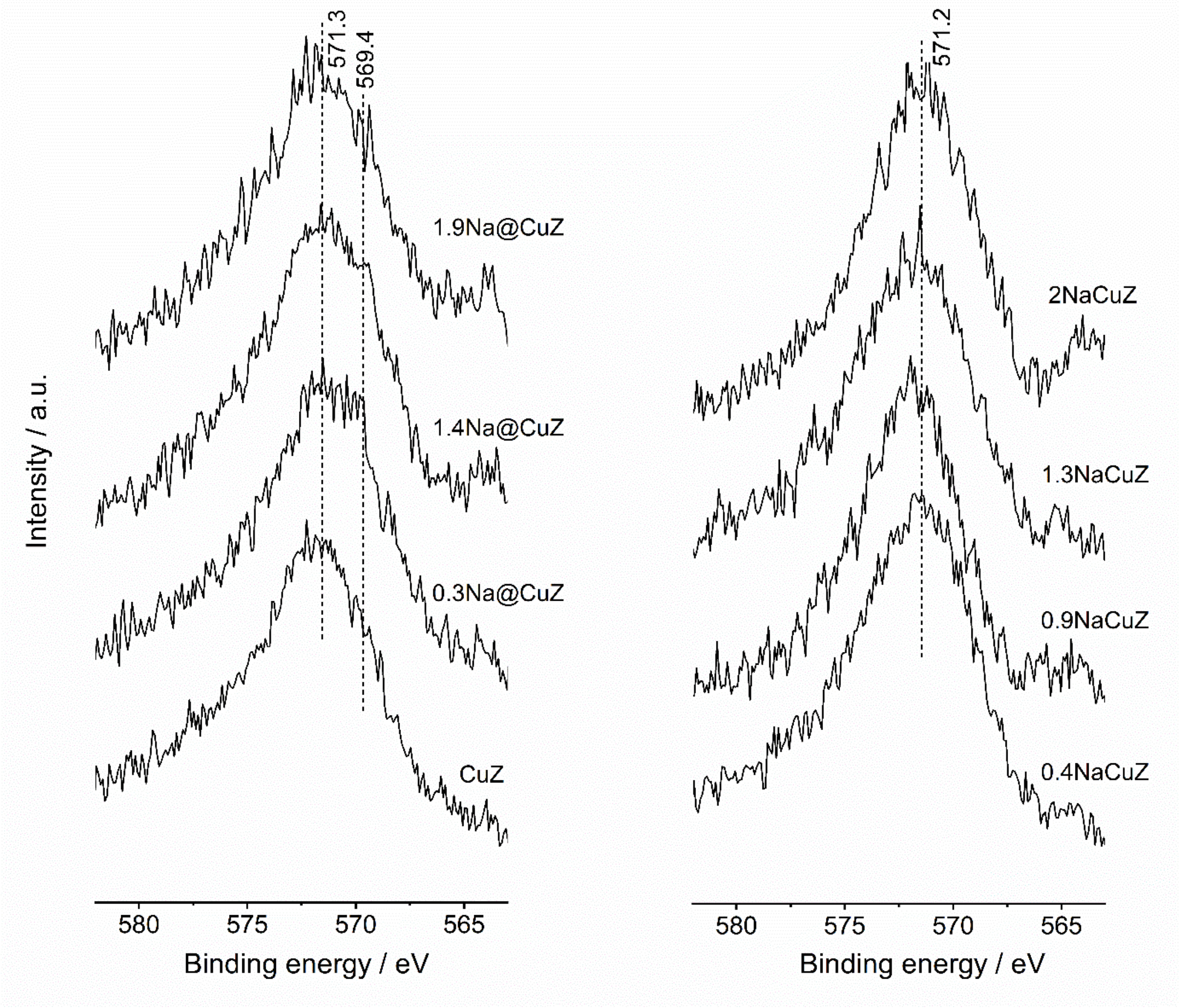

Figure S4. X-ray photoelectron spectroscopy Auger spectra of Cu LMM regions for the indicated freshly reduced Na-modified catalysts.

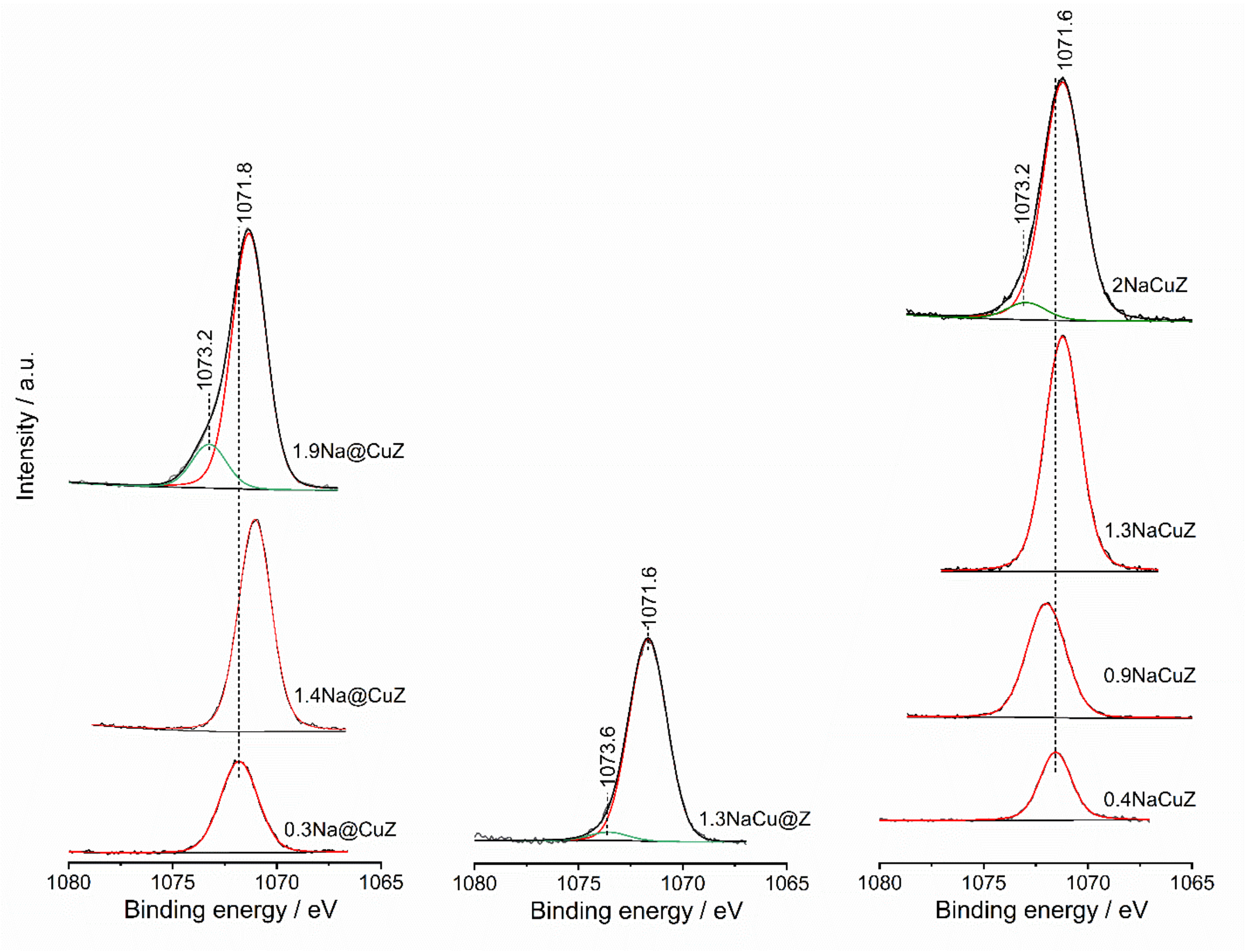

Figure S5. Na 1s XPS spectra of Na-modified catalysts prepared via different synthetic routes.

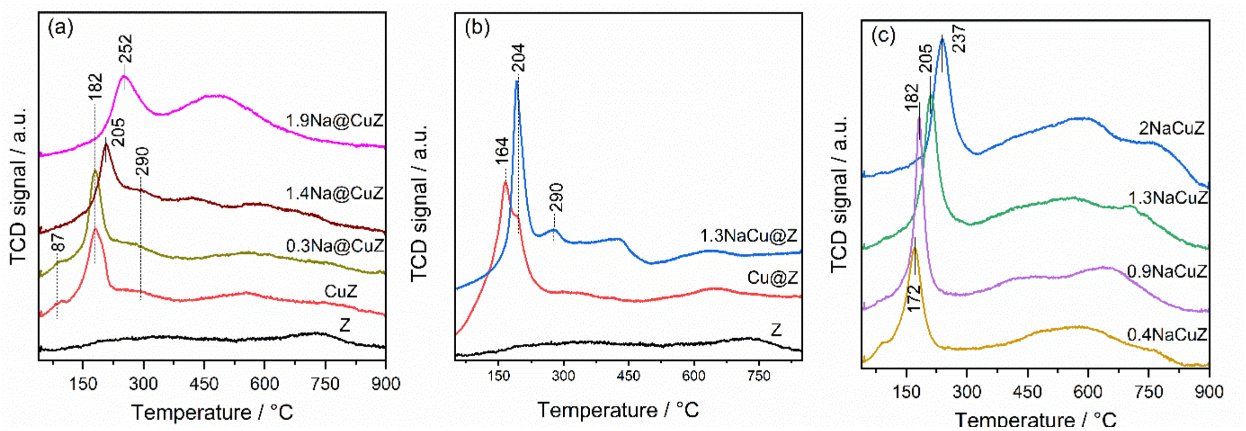

Figure S6. H_2-TPR profiles of (a) pure ZrO_2, CuZ, and xNa@CuZ samples (where x represents wt% Na). In these samples, CuZ was prepared via co-precipitation, and Na was added by wet impregnation; (b) xNaCu@Z samples (where x represents wt% Na) in which Cu and Na were

sequentially added on ZrO_2 via wet impregnation (Cu first, followed by Na) (the TPR profile of pure ZrO_2 is included for comparison); and (c) xNaCuZ samples (where x represents wt% Na) prepared using the one-pot synthesis method.

The TPR profile of pure ZrO_2 (**Figure S6a**) shows negligible reduction behavior. In contrast, the TPR profile of the CuZ sample exhibits reduction peaks at 87°C, 182°C, and 293°C, indicating a heterogeneous distribution of CuO_x species (**Figure S6a**). The addition of 0.3 wt% Na^+ (0.3Na@CuZ) has a negligible effect on the reduction behavior. However, as the Na^+ content increases, the main reduction peak shifts from 182°C to 205°C and 252°C for 1.4Na@CuZ and 1.9Na@CuZ, respectively. The addition of Na^+ does not affect the reduction peak at 290°C. The first reduction peak, which is affected by Na^+ addition, could be due to the reduction of dispersed CuO_x species, while the peak at 293°C, which remains unaffected, may correspond to Cu single sites strongly interacting with ZrO_2 or inaccessible Cu single sites incorporated in the ZrO_2 lattice. This assignment is supported by EPR results (see **Figure 4a** in the EPR section of the main manuscript), which show that the Cu species in/on these samples are predominantly dispersed as single sites.

On the other hand, samples in which Cu was added via wet impregnation (Cu@Z) show two reduction peaks at 164°C and 204°C (**Figure S6b**), attributed to the reduction of CuO_x clusters or small nanoparticles (NPs). The addition of Na^+ (in the 1.3NaCu@Z) eliminates the low-temperature reduction peak and leads to the appearance of another peak at 290°C. This new peak is possibly due to the slight agglomeration of small CuO_x species into bulk CuO nanoparticles after Na^+ addition, rather than the presence of Cu single sites. This interpretation is supported by both EPR and STEM results, which suggest the formation of CuO NPs.

In contrast, samples prepared via the one-pot approach exhibit a more homogeneous distribution of CuO_x species (**Figure S6c**), with the reduction peak at 172°C gradually shifting to higher temperatures as the Na^+ content increases.

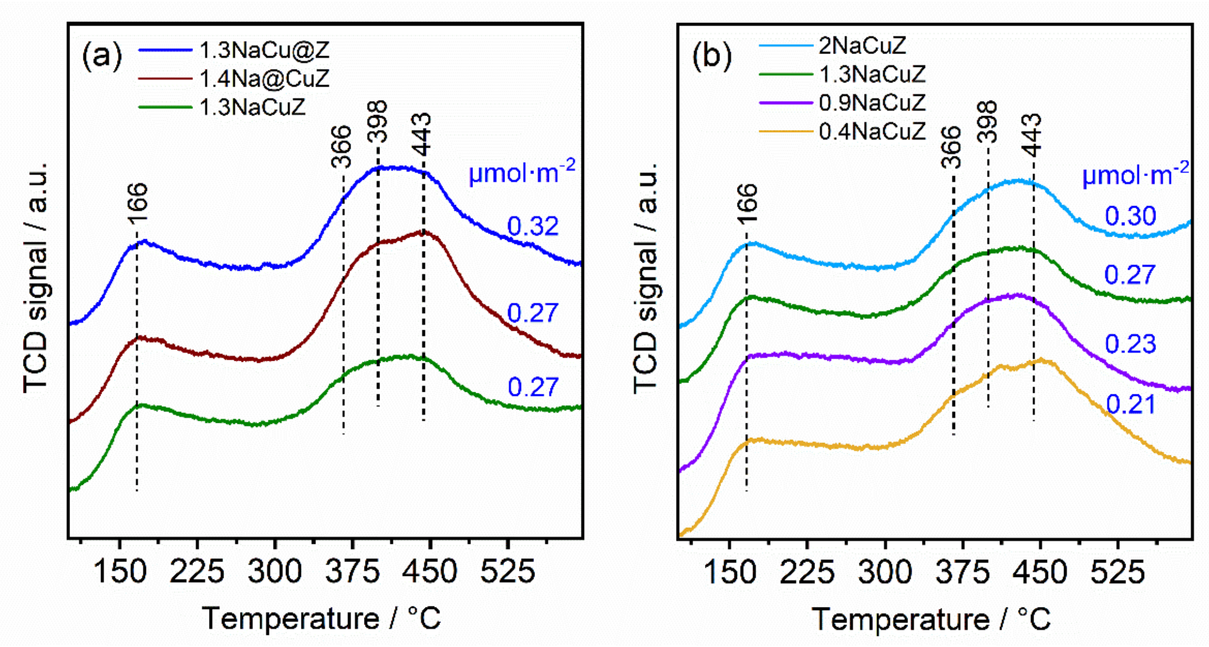

Figure S7. CO_2-TPD profiles of (a) Na-modified samples with similar Na contents but prepared using different synthetic routes; and (b) xNaCuZ catalysts synthesized via the one-pot method (x represents the wt% of Na). The blue numbers represent the amount of adsorbed CO_2 in μmol/m^2.

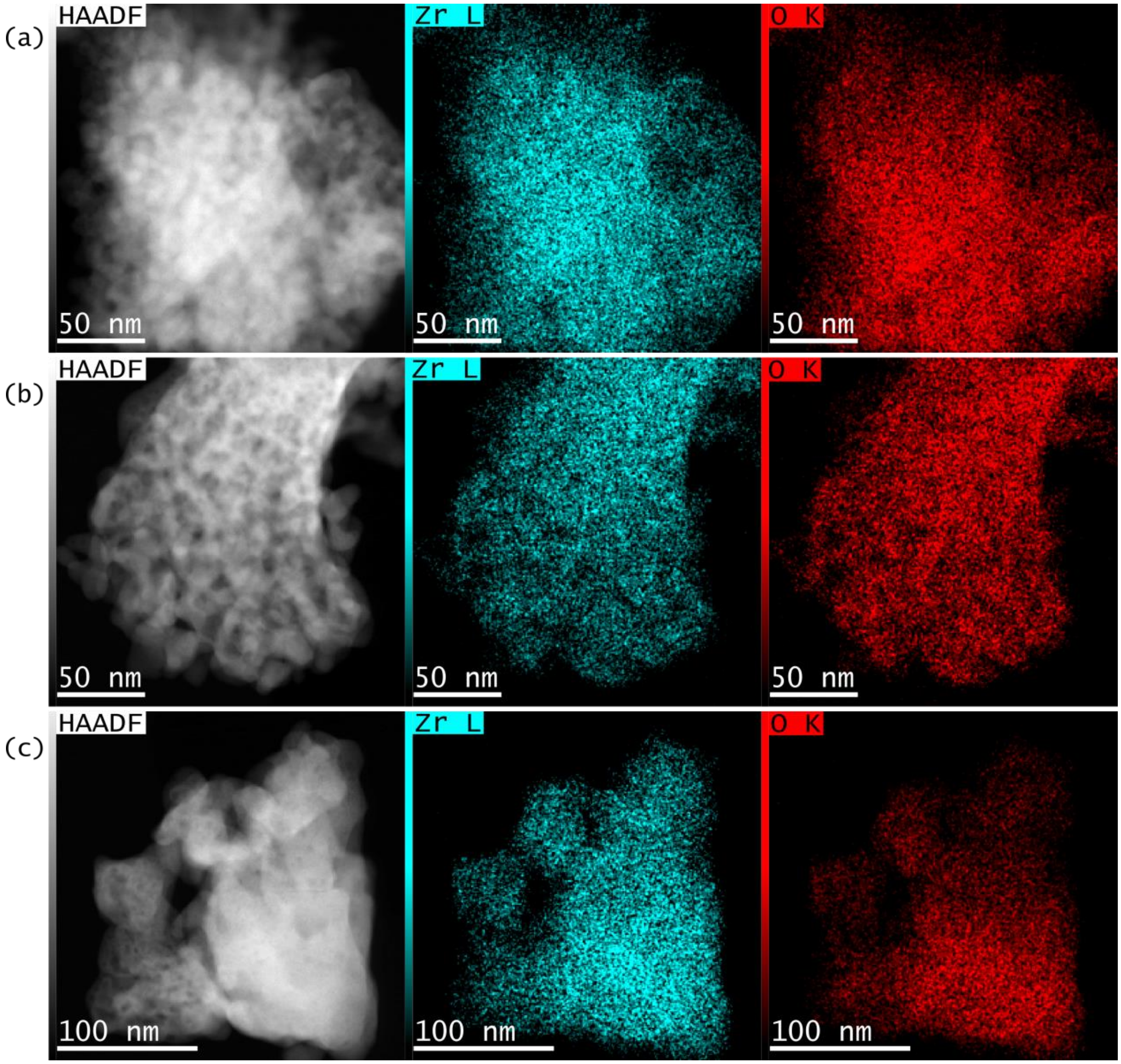

Figure S8. HAADF images and the corresponding EDX elemental mapping images of Zr and O for (a) 1.4Na@CuZ, (b) 1.3NaCu@Z, and (c) 1.3NaCuZ catalysts.

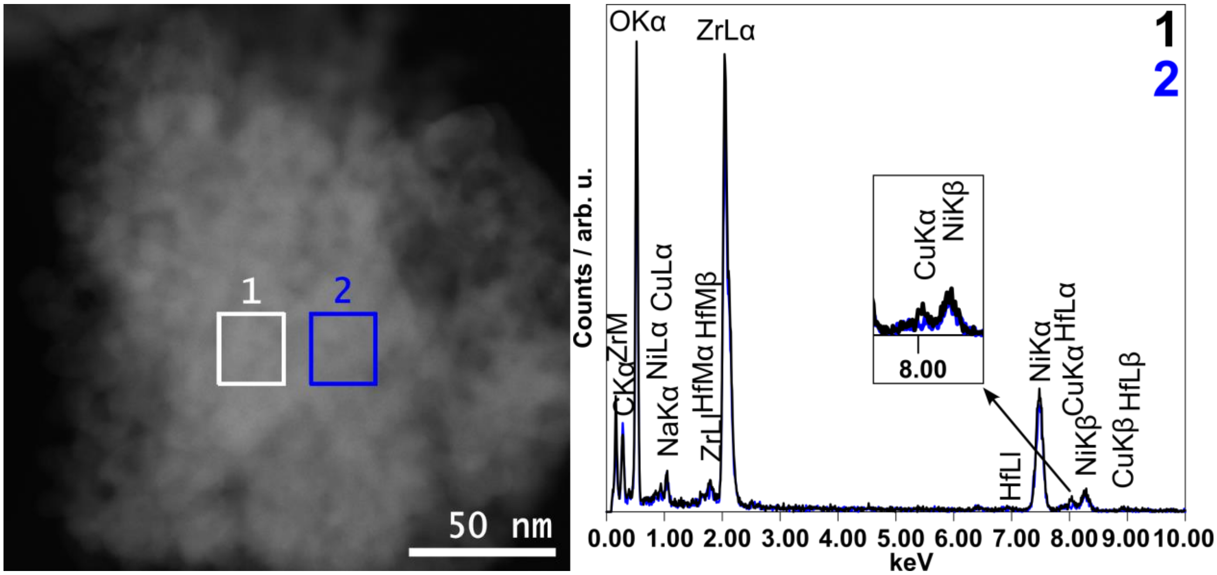

Figure S9. Selected EDX spectra (right) of the highlighted regions in the corresponding STEM-HAADF image (left) of 1.4Na@CuZ. The spectra are obtained from the elemental map measurement shown in **Figure 2a** and **Figure S8a**.

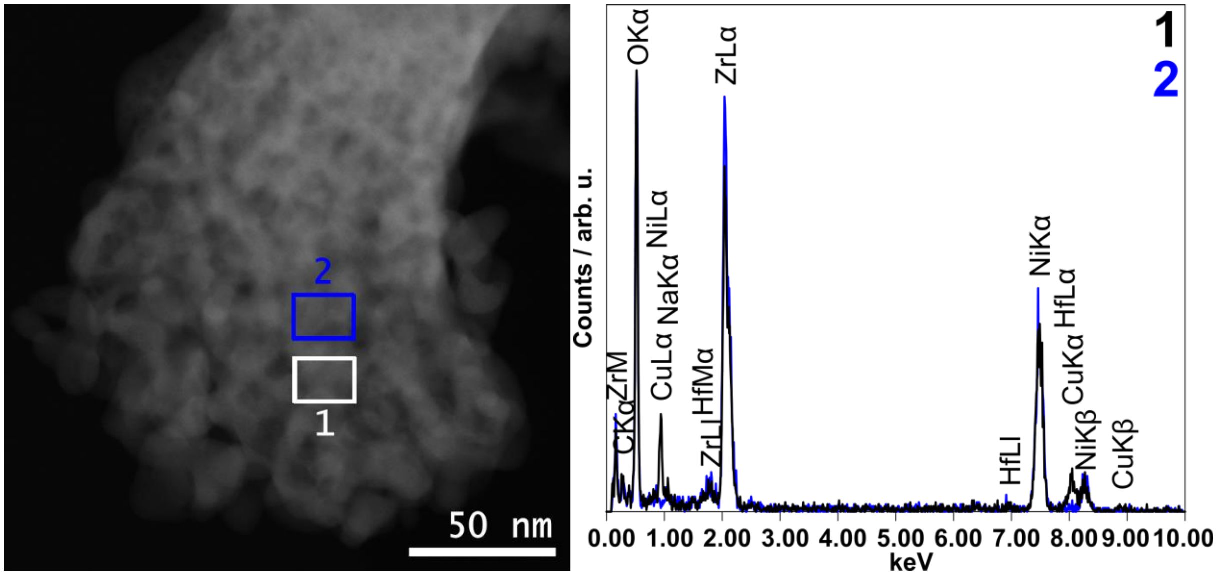

Figure S10. Selected EDX spectra (right) of the highlighted regions in the corresponding STEM-HAADF image (left) of 1.3NaCu@Z. The spectra are obtained from the elemental map measurement shown in **Figure 2b** and **Figure S8b**.

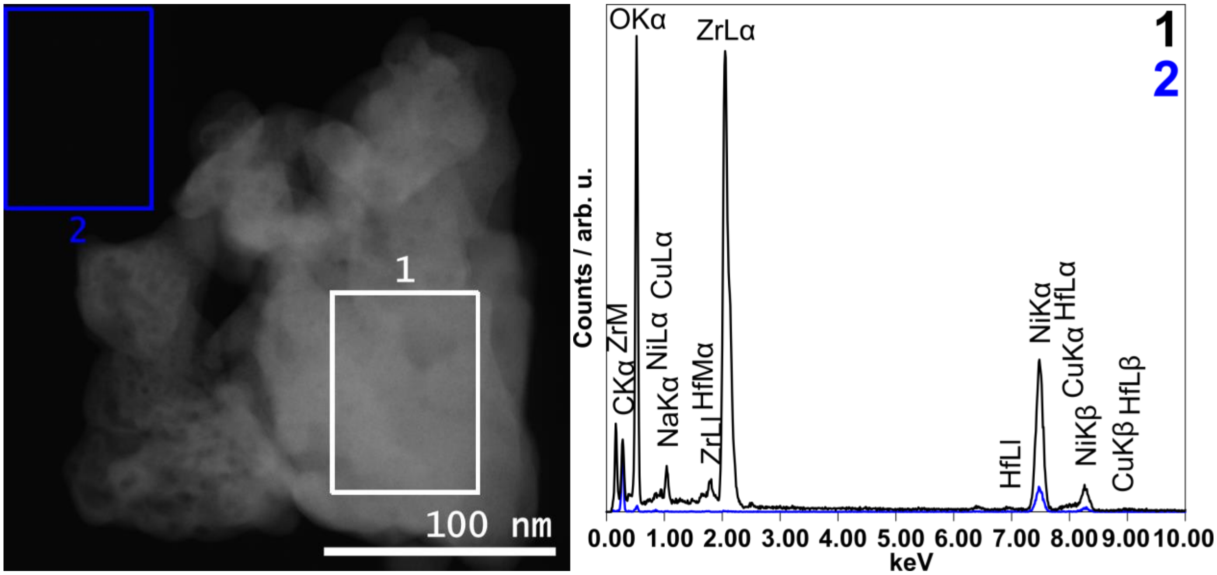

Figure S11. Selected EDX spectra (right) of the highlighted regions in the corresponding STEM-HAADF image (left) of 1.3NaCuZ. The spectra are obtained from the elemental map measurement shown in **Figure 2c** and **Figure S8c**.

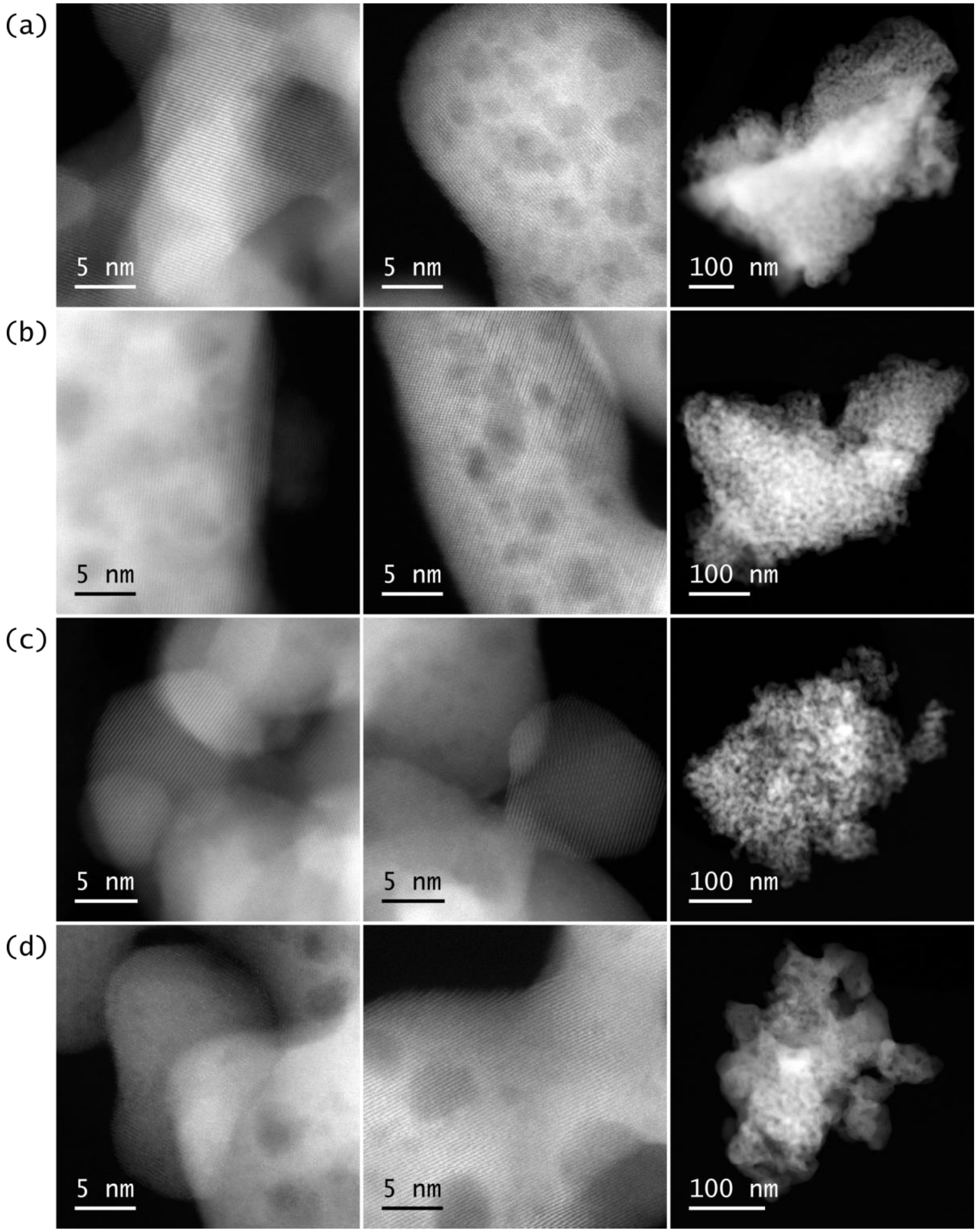

Figure S12. Selected STEM-HAADF images at different magnifications for (a) CuZ, (b) 1.4Na@CuZ, (c) 1.3NaCu@Z, and (d) 1.3NaCuZ catalysts.

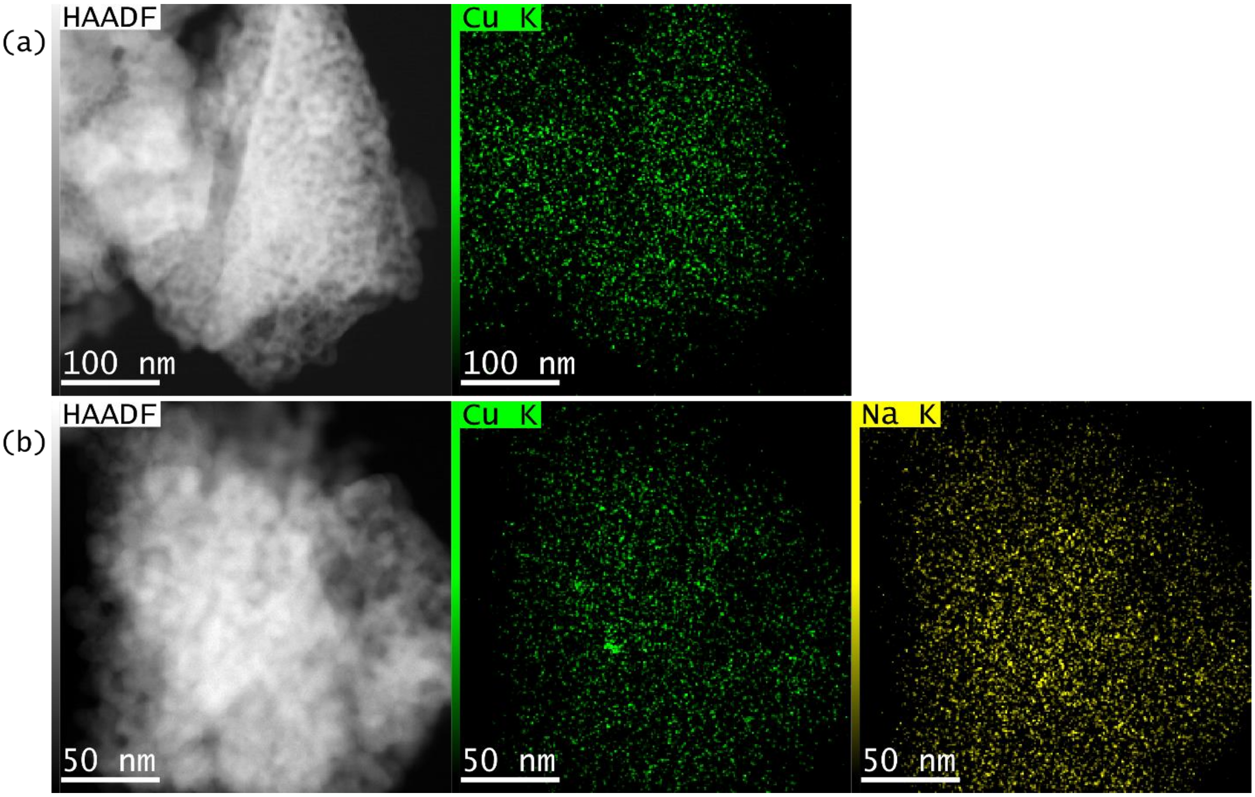

Figure S13. HAADF image and the corresponding EDX elemental mapping images of Cu and Na for (a) CuZ, and (b) 1.4Na@CuZ.

(a)

(b)

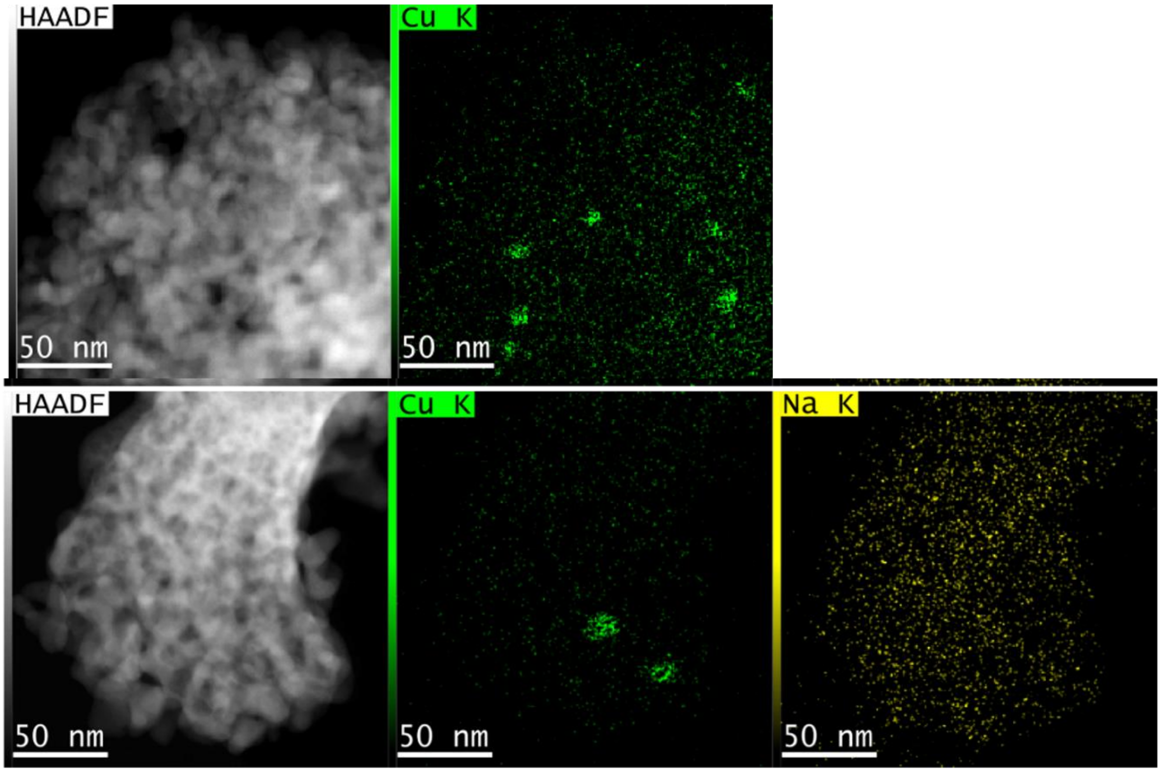

Figure S14. HAADF image and the corresponding EDX elemental mapping images of Cu and Na for (a) Cu@Z, and (b) 1.3NaCu@Z.

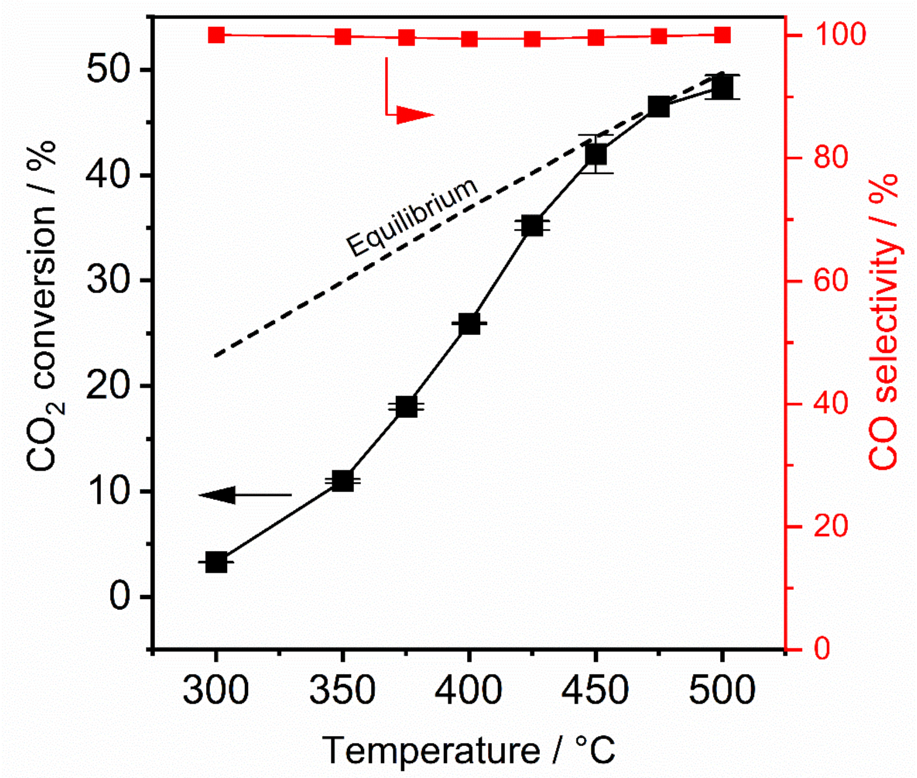

Figure S15. CO_2 conversion over 1.3NaCuZ catalyst between 300-500 °C.

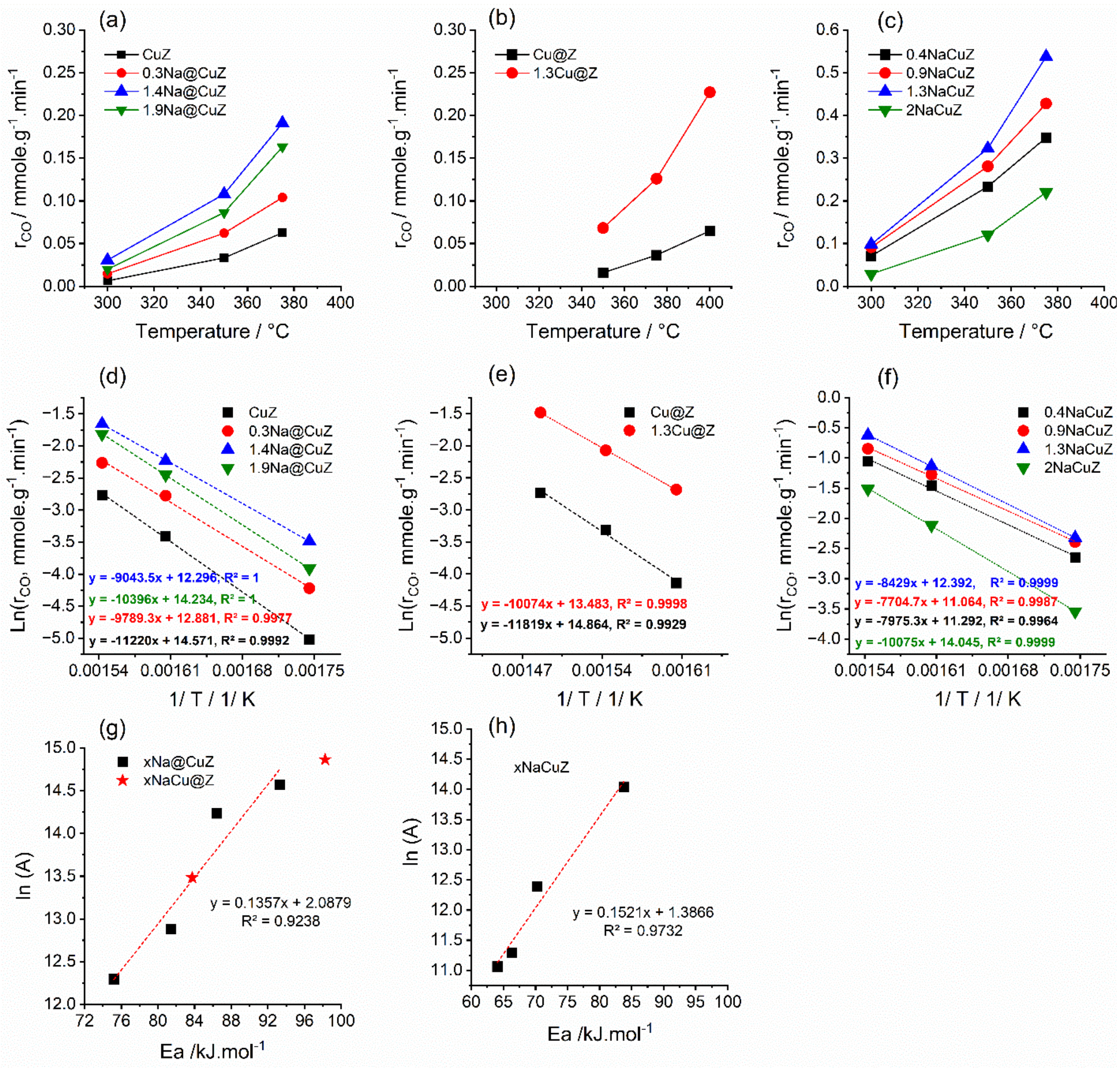

Figure S16. CO formation rates over: (a) CuZ and xNa@CuZ (Na added via wet impregnation to co-precipitated CuZ); (b) xNaCu@Z (Cu and Na sequentially added to ZrO_2 via wet impregnation); (c) xNaCuZ (one-pot synthesis with x wt% Na). (d–f) show the corresponding Arrhenius plots. (g) and (h) display evidence of an approximate compensation effect based on the correlation between ln A and activation energy (E_a) for xNa@CuZ and xNaCuZ catalysts, respectively. The two red stars in panel (g) represent the xNaCu@Z catalysts.

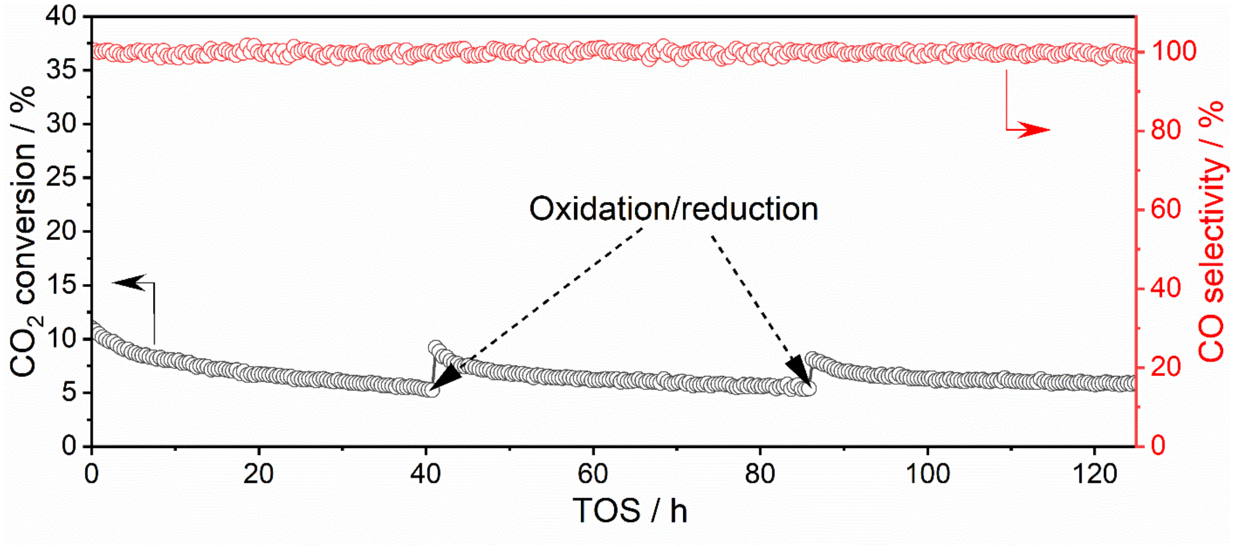

Figure S17. Stability test of 1.3NaCuZ for ~125 h at 350°C with a space velocity of 24,000 $mL \cdot g_{cat}^{-1} \cdot h^{-1}$, with re-activation by oxidation (5% O_2/N_2) and reduction (50% H_2/N_2) at 20 mL/min flow for 30 min at the same test temperature.

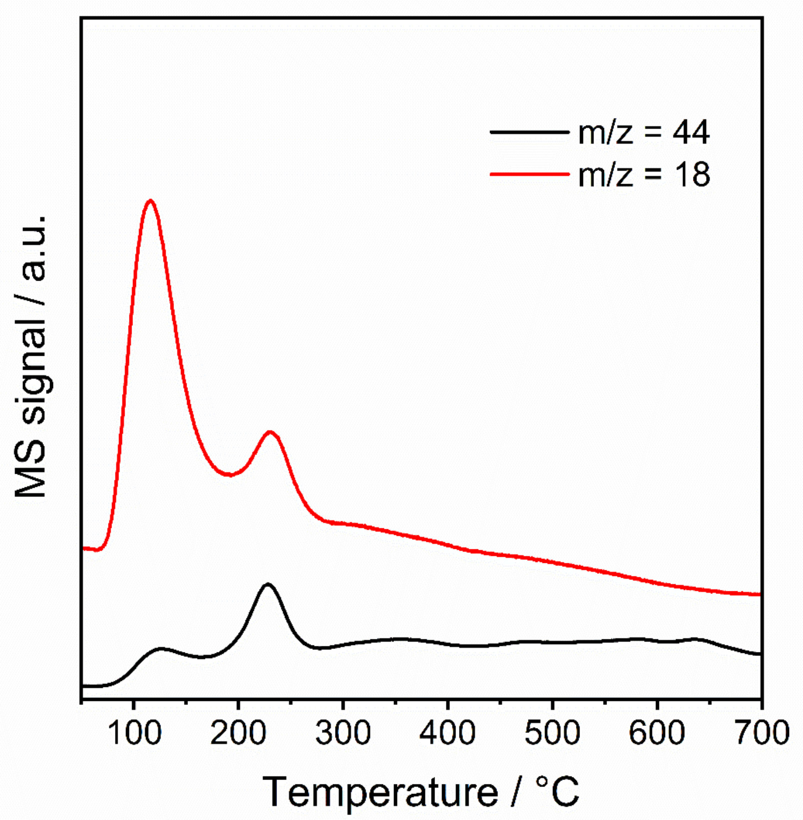

Figure S18. TPO profile of the spent 1.3NaCuZ catalyst after a 45-h on-stream stability testing at 400 °C.

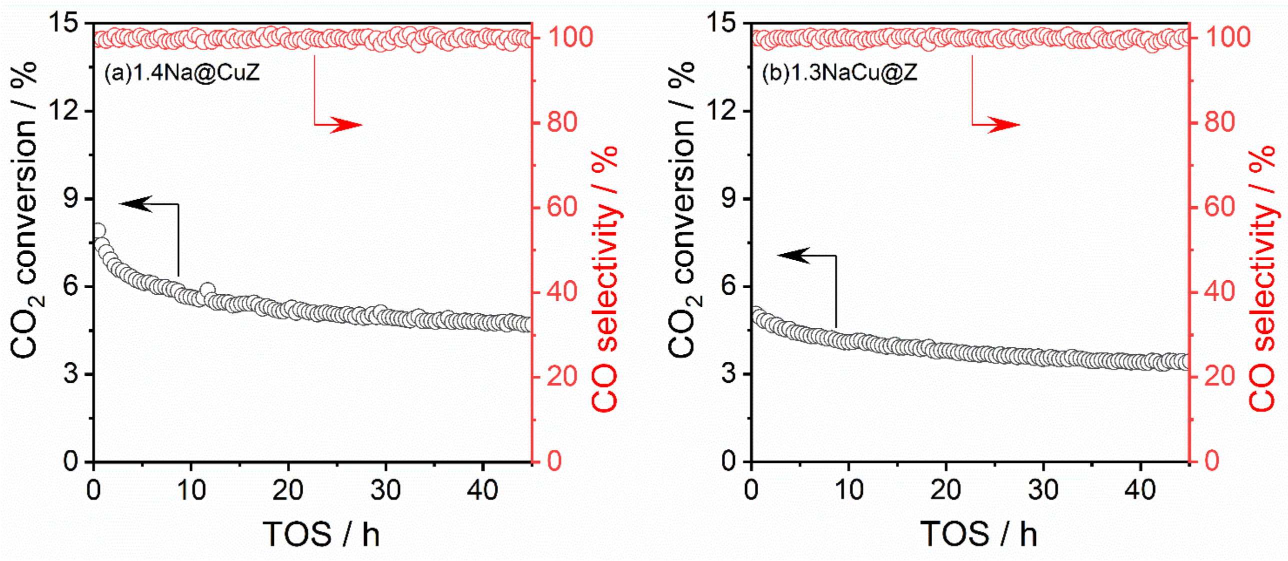

Figure S19. Stability tests for ~ 45 h at 350°C with a space velocity of 12,000 mL·g_{cat}^{-1}·h^{-1}

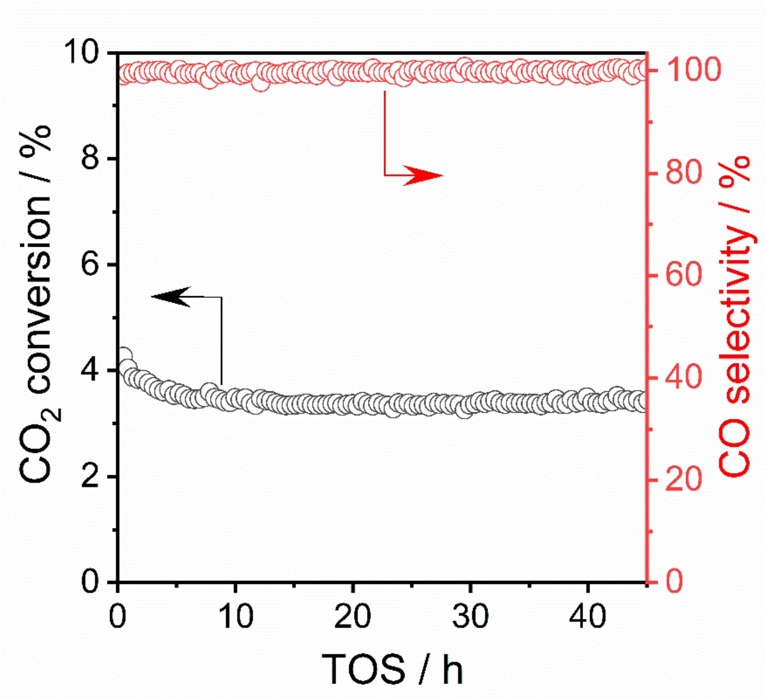

Figure S20. Stability test of CuZ for ~ 45 h at 350°C with a space velocity of 6,000 mL·g_{cat}^{-1}·h^{-1}

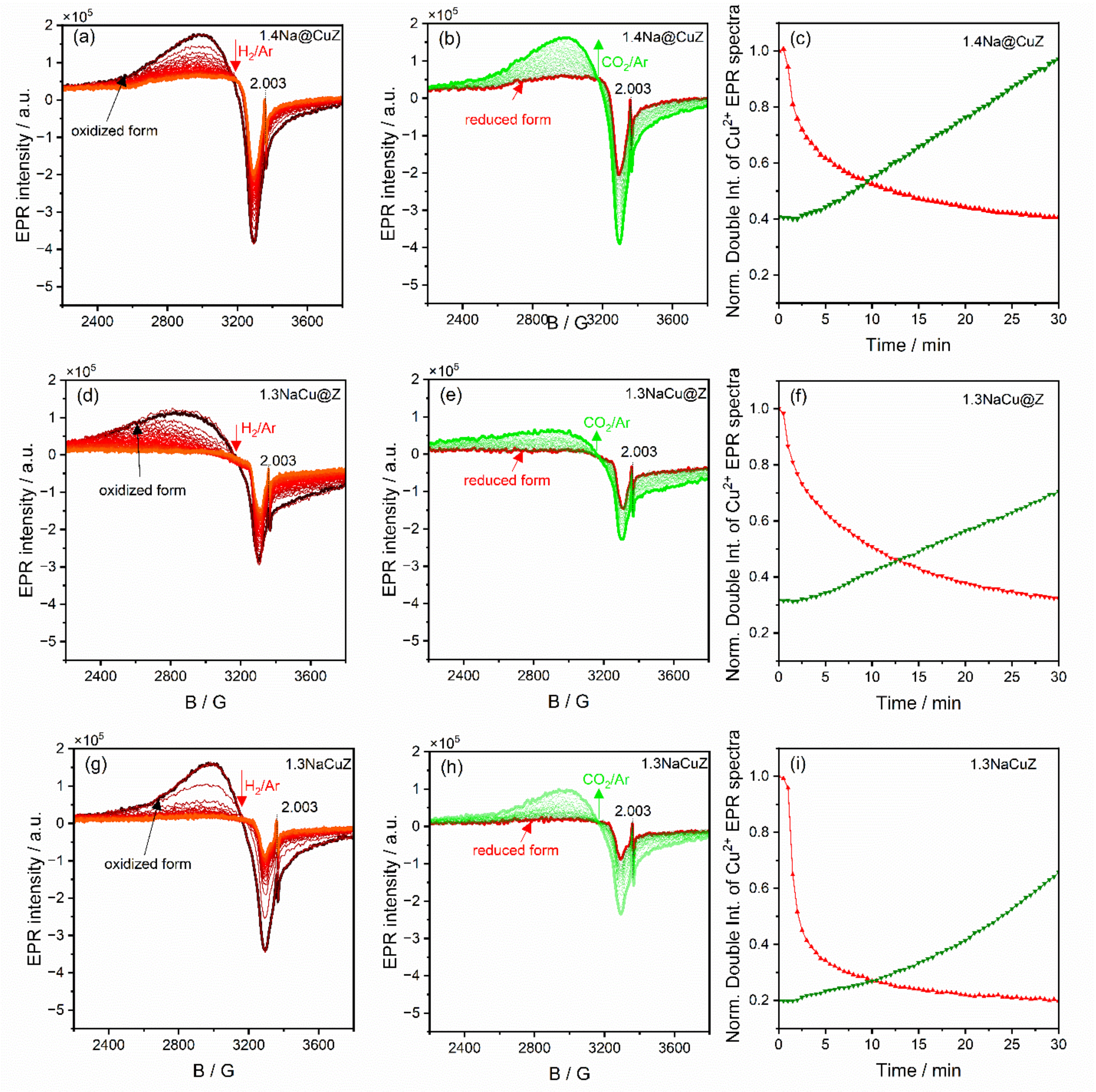

Figure S21. In-situ EPR spectra recorded at 350 °C: (a, d, g) for the oxidized form (black line) during a 30-min reduction in a 50% H_2/Ar flow; (a, e, h) for the reduced form (red line) during a 30-min oxidation in a 15% CO_2/Ar flow; and (c, f, i) the corresponding normalized double integral of the Cu^{2+} EPR spectra recorded at 350 °C under a 50% H_2/Ar flow (red line) and a 15% CO_2/Ar flow (green line) over time.

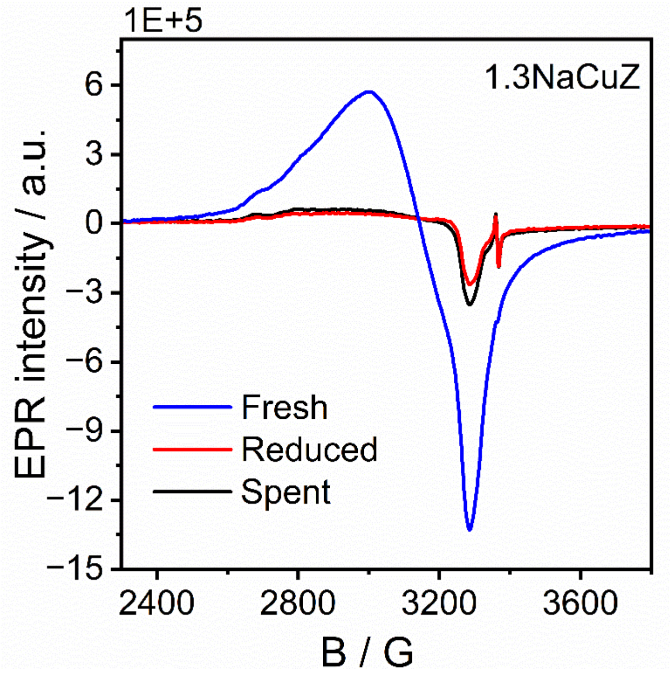

Figure S22. In-situ EPR spectra at 300 K for fresh oxidized, reduced, and spent (after 15 h) forms of 1.3NaCuZ.

All measurements in **Figure S22** were performed at 300 K because the spent catalyst was measured under the RWGS reaction mixture (CO_2 + H_2) and measuring at -173 °C would cause the condensation of CO_2, making it impractical. Then, we also repeated the measurements for the fresh and reduced forms at 300 K for better comparison with the spent form.

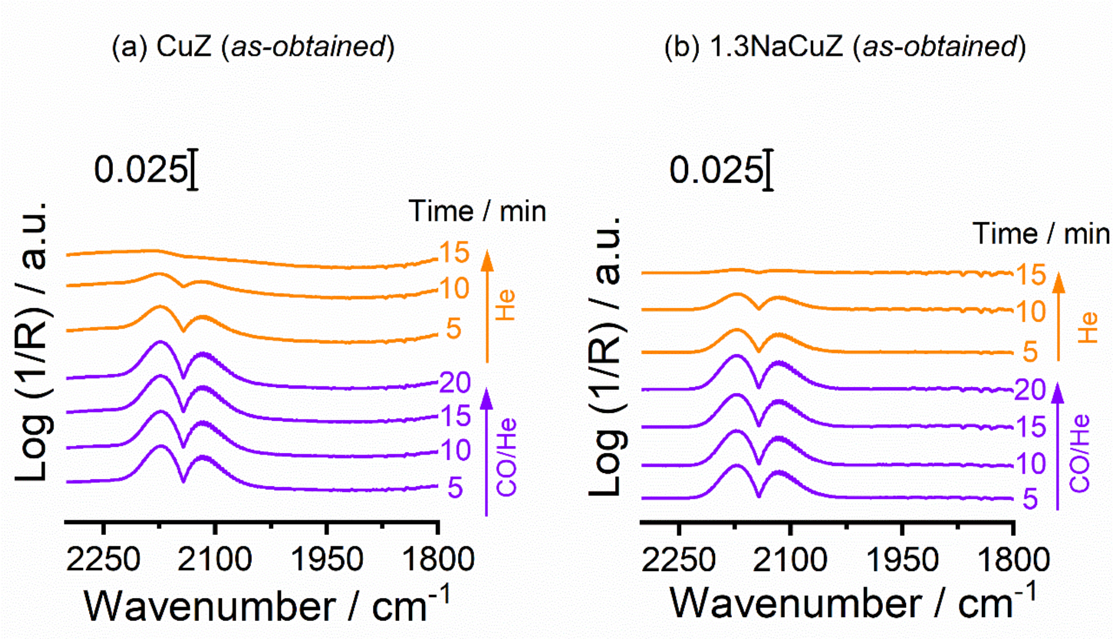

Figure S23. Time-resolved in situ CO-DRIFTS spectra collected at 20 °C for (a) CuZ and (b) 1.3NaCuZ catalysts under a flow of 1% CO/He for 20 min (shown in violet), followed by 100% He for 15 min (shown in orange), after preheating the catalysts at 400 °C under He for 1 h.

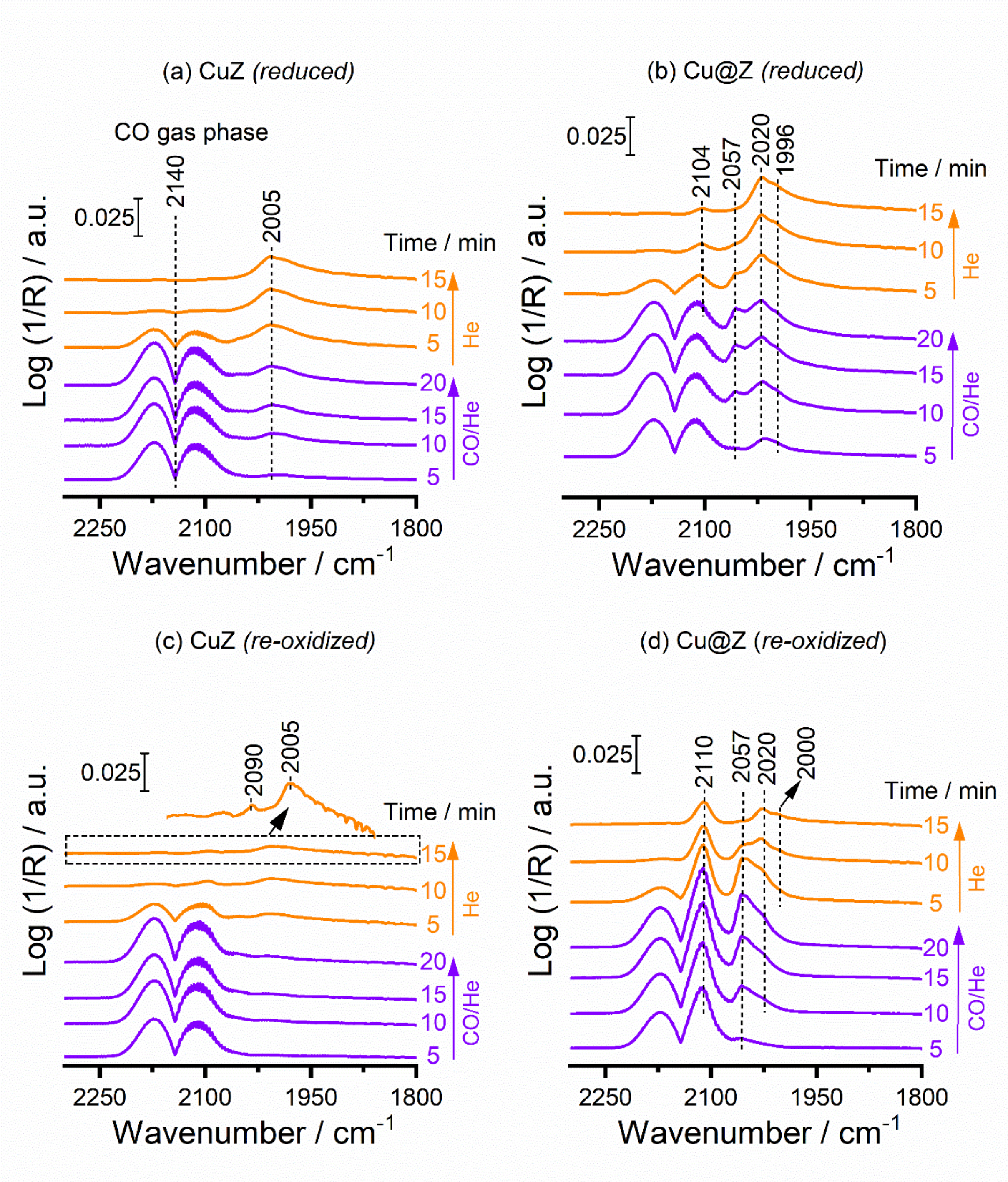

Figure S24. Time-resolved in-situ CO-DRIFT spectra over Na-free catalysts prepared by two methods: co-precipitation (CuZ) and wet impregnation (Cu@Z). Spectra were recorded at 20 °C under a 1% CO/He flow for 20 min (violet) followed by a 100% He flow for 15 min (orange). Panels (a) and (b) show spectra for catalysts in their reduced state, pretreated at 400 °C under a 50% H_2/He flow for 1 h, while panels (c) and (d) displays spectra for the re-oxidized state,

pretreated at 350 °C under a 15% CO_2/He flow for 30 min. A background spectrum in flowing He at 20 °C was subtracted from all spectra recorded in the CO-containing atmosphere.

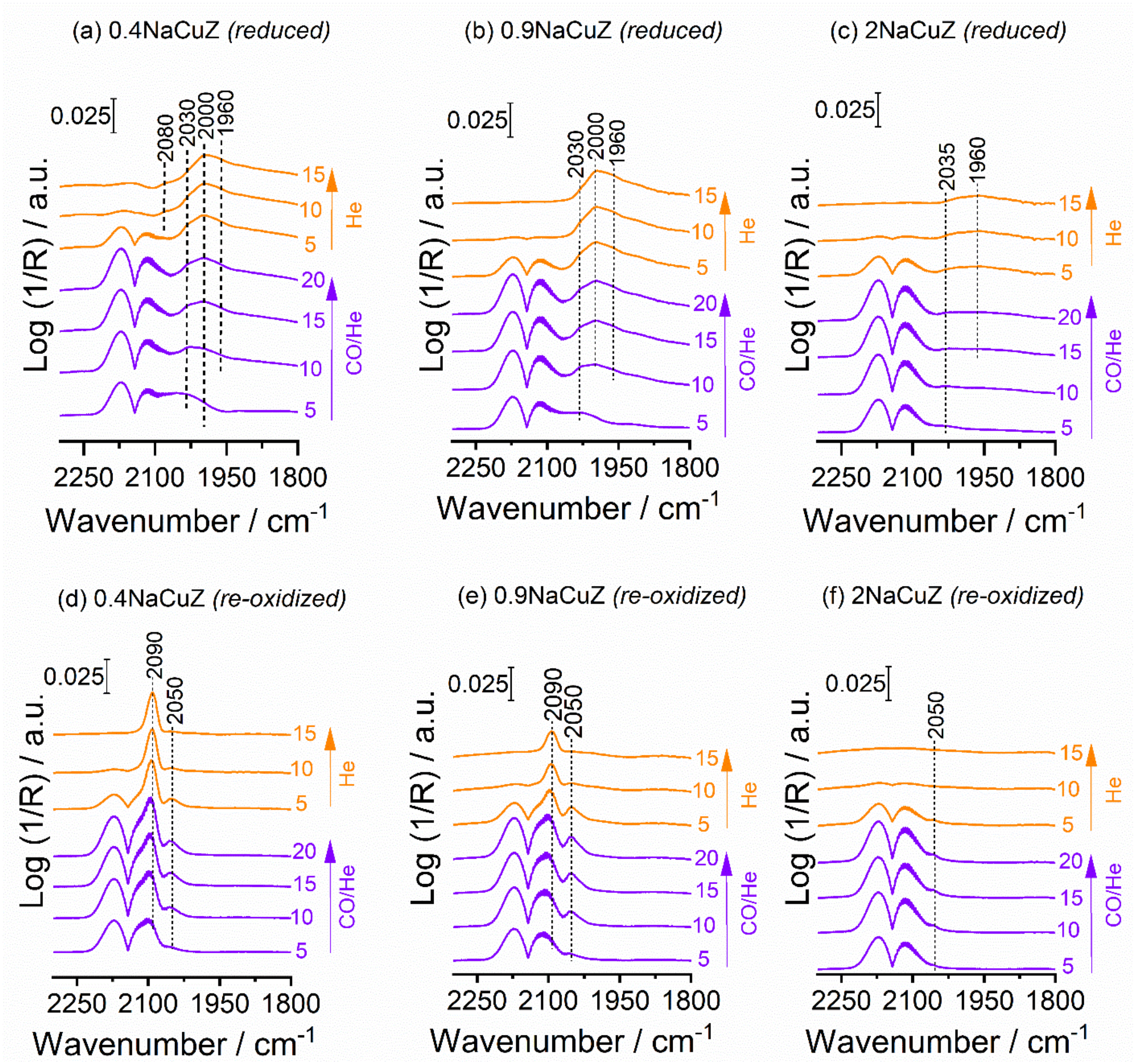

Figure S25. Time-resolved in-situ CO-DRIFT spectra of Na-modified catalysts prepared by the one-pot method (xNaCuZ) with varying Na content (x). Spectra were recorded at 20 °C under a 1% CO/He flow for 20 min (violet), followed by a 100% He flow for 15 min (orange). Panels (a-c) show spectra for catalysts in the reduced state, pretreated at 400 °C under a 50% H_2/He flow for 1 h, while panels (d-f) show spectra for the re-oxidized state, pretreated at 350 °C under a 15% CO_2/He flow for 30 min. A background spectrum collected in flowing He at 20 °C was subtracted from all spectra recorded in the CO-containing atmosphere.

CO adsorption on H_2-reduced CuZ (**Figure S24a**) shows a band at 2005 cm^{-1}, corresponding to bridge-bonded CO species on Cu^0 sites.[1-2] while the CO_2-reoxidized form (**Figure S24c**) exhibits the same bands with reduced intensity, along with an additional band at 2090 cm^{-1}, attributed to CO adsorbed on Cu^+ sites.[3-5] In contrast, CO adsorption on H_2-reduced Cu@Z (**Figure S24b**) shows several bands at 2104 cm^{-1}, 2057 cm^{-1}, 2020 cm^{-1}, and 1996 cm^{-1}. The band at 2104 cm^{-1} is attributed to CO adsorbed on Cu^+ sites, [3-5] while the band at 2057 cm^{-1} corresponds to CO linearly adsorbed on Cu^0. The bands at 2020 cm^{-1} and 1996 cm^{-1} are assigned to bridge-bonded CO species on Cu^0 sites.[1-2] It worth mentioning that the 2057 cm^{-1} band is only detectable in the presence of gaseous CO (indicated by the doublet at 2140 cm^{-1}) and is easily removed by purging with He. We suggest that the 2057 cm^{-1} band is likely due to CO adsorption on Cu^0 in close proximity to Cu^+ sites. The increase in the intensity of this band on its CO_2-reoxidized form (**Figure S24d**) supports this hypothesis, as the intensity of the CO-Cu^+ band (2104 cm^{-1}) significantly increases compared to the H_2-reduced form. The presence of multiple CO adsorption bands, CO–Cu^+ at 2090 cm^{-1} and CO-Cu^0 between 2000-2057 cm^{-1}, after exposing the Cu@Z catalyst to CO_2 (**Figure S24d**) indicates that the re-oxidation of Cu sites (Cu^0 to Cu^{2+}) occurs more slowly compared to CuZ. These results suggest that, although CuZ has a lower surface Cu density (as indicated by band intensity, **Figure S24a** versus **S24b**), its surface Cu sites exhibit higher redox activity than those on Cu@Z. This aligns well with the slightly higher RWGS activity observed for CuZ compared to Cu@Z (**Figure 3d** in the manuscript).

All H_2-reduced Na-modified catalysts prepared using the one-pot approach, except 2NaCuZ, showed high intensity of CO bands (**Figure S25**). The very weak adsorption of CO on the 2NaCuZ catalyst (**Figure S25c**) can be explained by the extremely low sticking coefficient of CO on metal surfaces saturated and/or covered with alkali metals.[6] This explains the lower RWGS activity of this catalyst (**Figure 3c** in the manuscript). For the 0.4NaCuZ and 0.9NaCuZ catalysts, both exhibit CO adsorption bands corresponding to Cu^+ (2090 cm^{-1}) and Cu^0 (2050 cm^{-1}). However, when comparing the CO band intensities between the H_2-reduced and CO_2-treated forms, the CO band intensity on 0.9NaCuZ (**Table S4**) is lower than that on 0.4NaCuZ (**Table S4**). This suggests that 0.9NaCuZ has higher redox activity, which is consistent with its higher RWGS performance (**Figure 3c** in the manuscript).

When comparing the CO adsorption over Na-modified catalysts with similar Na content (**Figure 5a-c**, in the manuscript). We found that 1.3NaCuZ and 1.4Na@CuZ catalysts displayed additional bands in the low-frequency region between 1900 and 1913 cm^{-1}. Dubois et al.[7-8] attributed this band to new adsorption sites or the direct interaction of CO with alkali via 1π rehybridization, [7-8] while Kappers et al. suggested that the ion-dipole interaction between the oxygen atoms of linearly adsorbed CO and alkali ions is responsible for the low-frequency shift, which could reach 100 cm^{-1}.[9] In general, the decrease in the frequency of the CO band indicates a stronger interaction between CO and the metal sites. This strong adsorption could be due to enhanced back-donation resulting from the presence of alkali and/or an increase in the number of Cu sites with a higher degree of unsaturation (e.g., highly dispersed small clusters).[10] Although Na^+ exists in the 1.3NaCu@Z catalyst, this low-frequency band was not observed. This suggests that the band is more likely due to the presence of defective Cu sites in close proximity to Na^+, which form in higher density in 1.3NaCuZ, based on the intensity of this band.

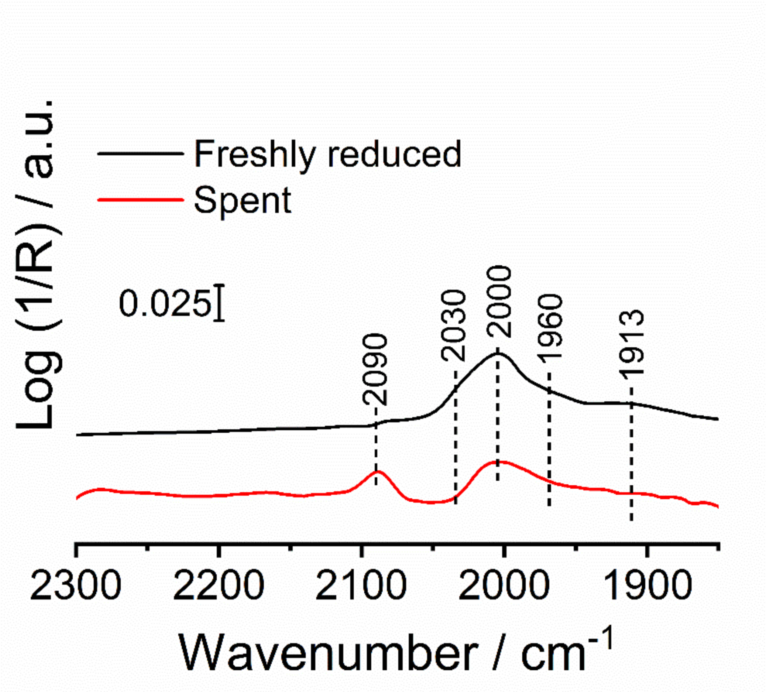

Figure S26. In-situ CO-DRIFTS spectra of freshly reduced and spent 1.3NaCuZ, recorded at 20 °C after exposure to a flow of 1% CO/He for 15 min, followed by 100% He for 10 min.

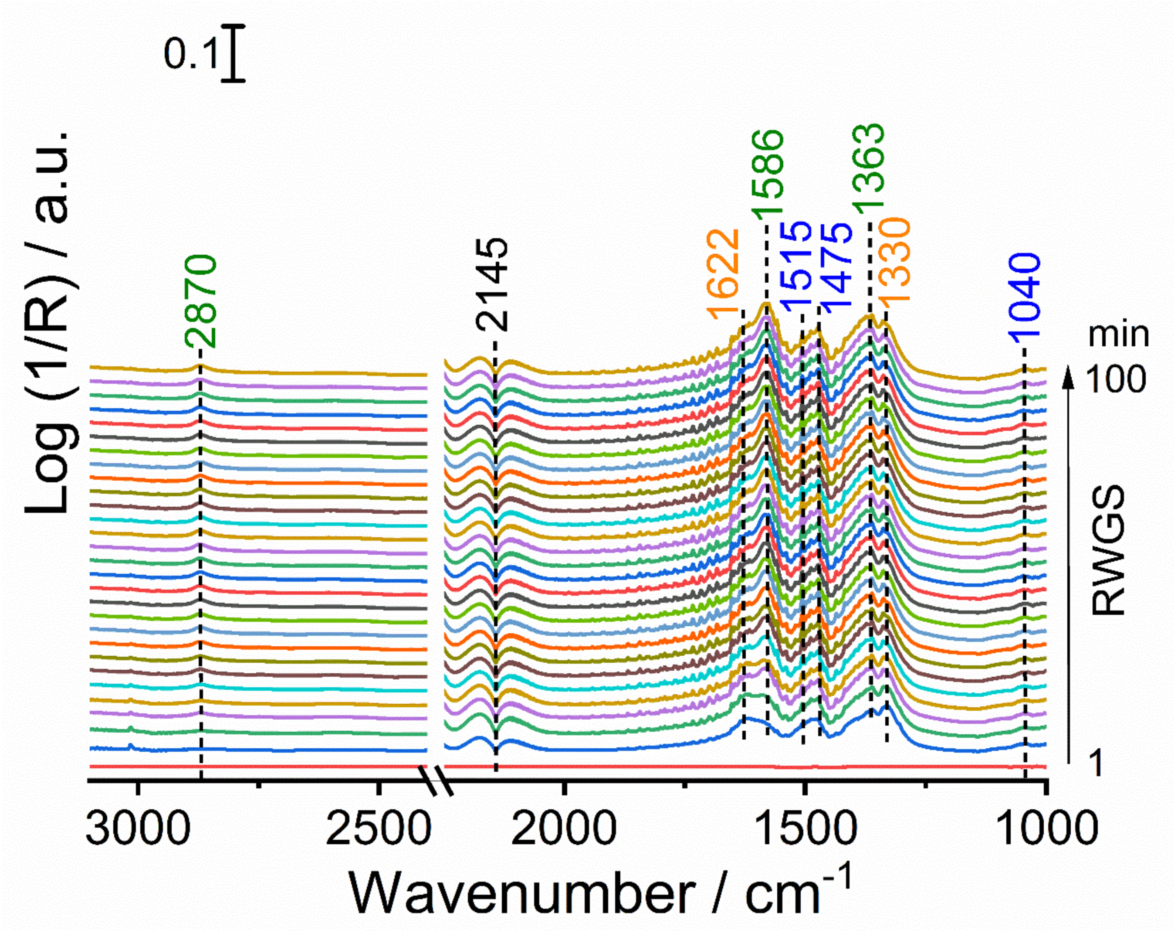

Figure S27. In situ time-resolved DRIFTS spectra of the RWGS reaction over CuZ collected up to 100 min. Reaction conditions: p = 1 bar, T = 350 °C, H_2/CO_2 = 3:1, total flow rate = 20 mL·min^{-1}. The first 10 spectra were recorded at 1-min intervals, followed by spectra taken every 5 min up to 100 min.

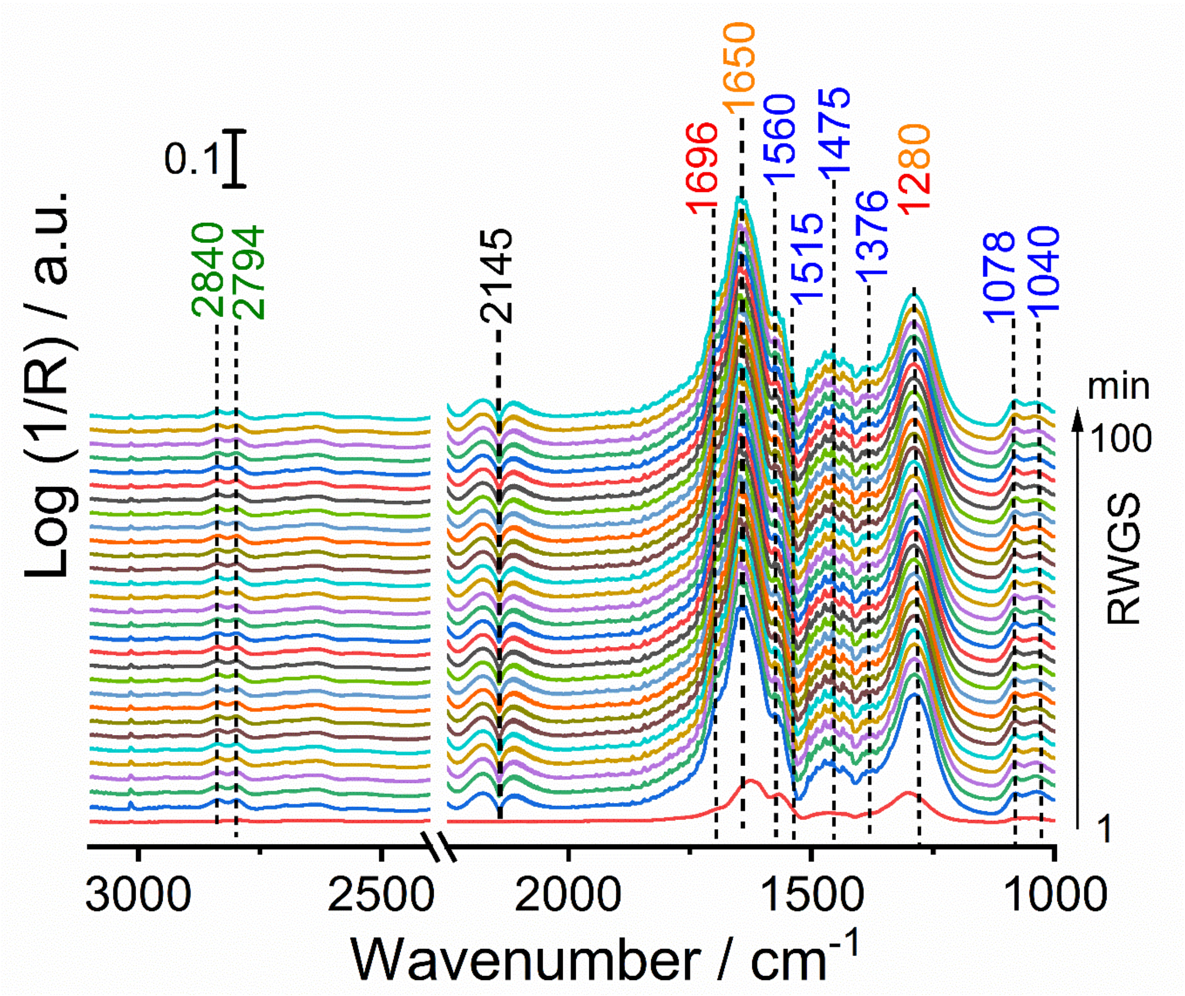

Figure S28. In situ time-resolved DRIFTS spectra of the RWGS reaction over 1.3NaCuZ collected over 100 min. Reaction conditions: p = 1 bar, T = 350 °C, H_2/CO_2 = 3:1, total flow rate = 20 mL·min^{-1}. The first 10 spectra were recorded at 1-min intervals, followed by spectra taken every 5 min up to 100 min.

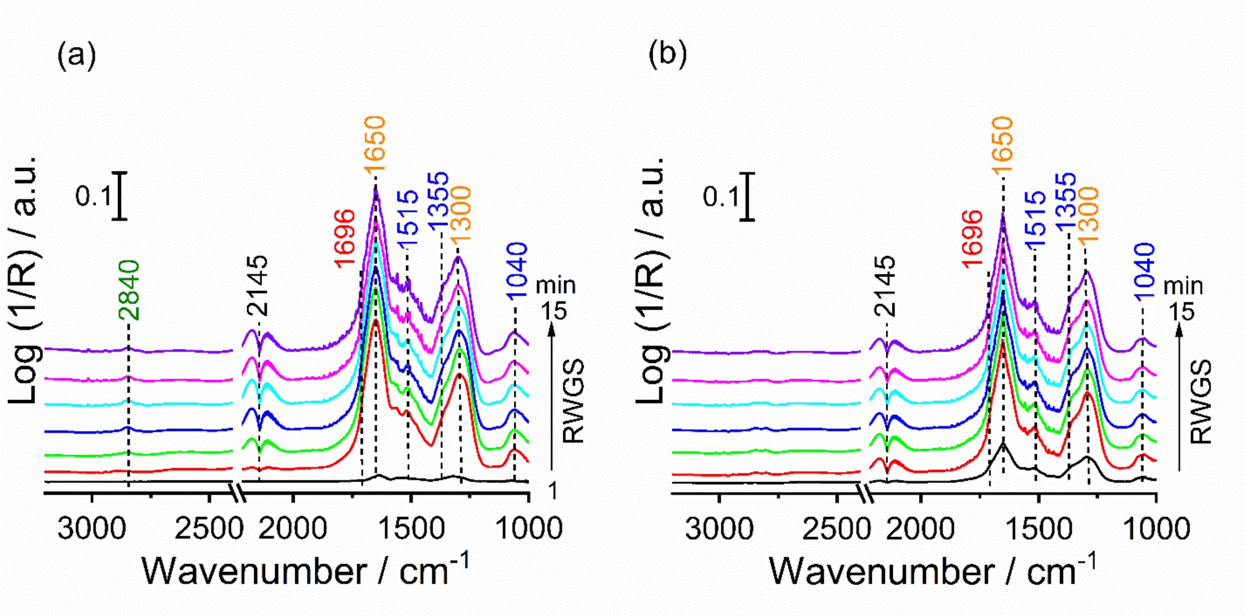

Figure S29. In-situ time-resolved DRIFT spectra of the RWGS reaction over (a) 1.4Na@CuZ and (b) 1.3NaCu@Z. Reaction conditions: p = 1 bar, T = 350 °C, H_2: CO_2 = 3:1; total flow rate = 20 mL·min^{-1}.

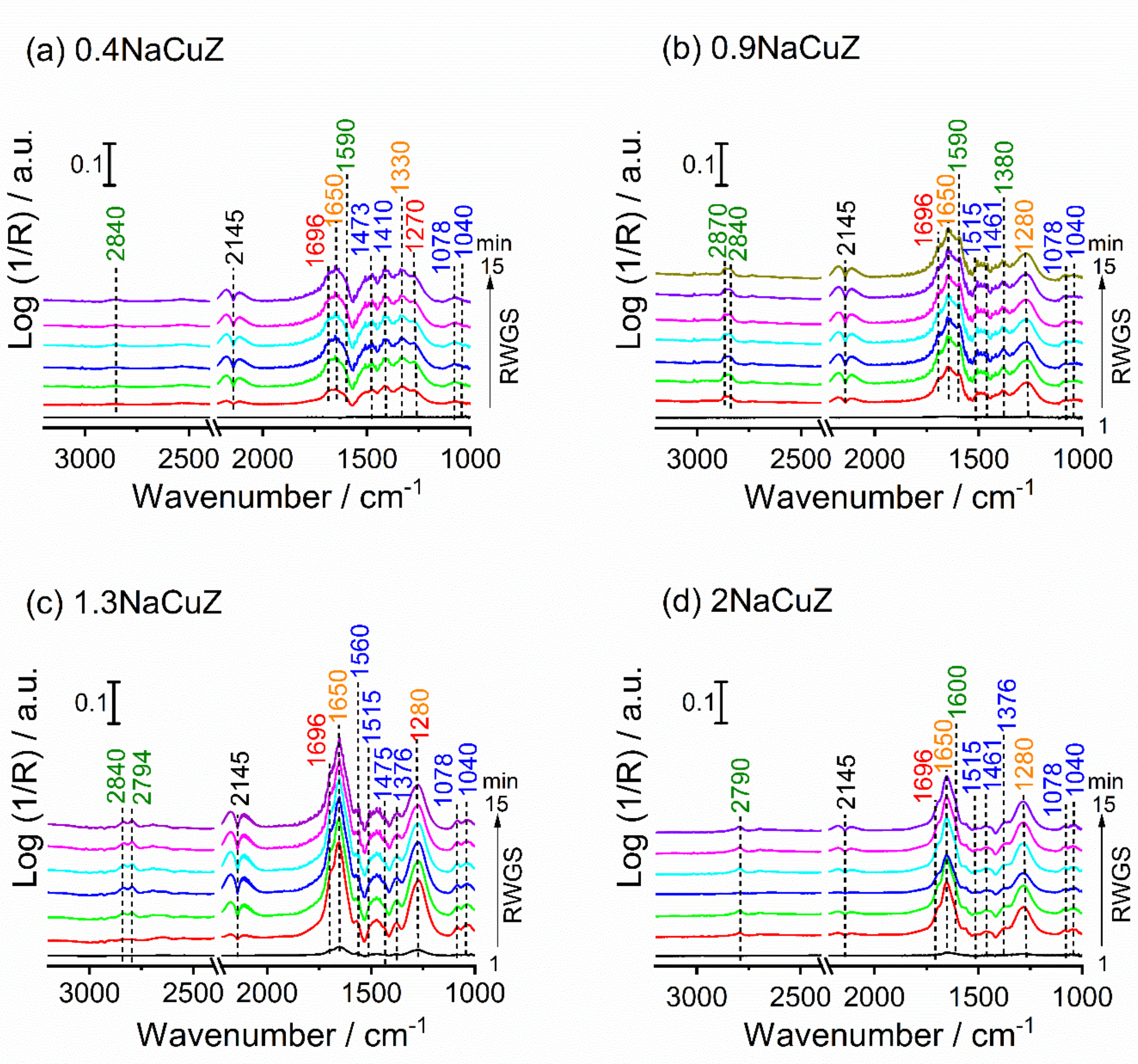

Figure S30. In-situ time-resolved DRIFT spectra of the RWGS reaction over the one-pot synthesized catalysts with different Na contents. Reaction conditions: p = 1 bar, T = 350 °C, H_2: CO_2 = 3:1; total flow rate = 20 mL·min^{-1}.

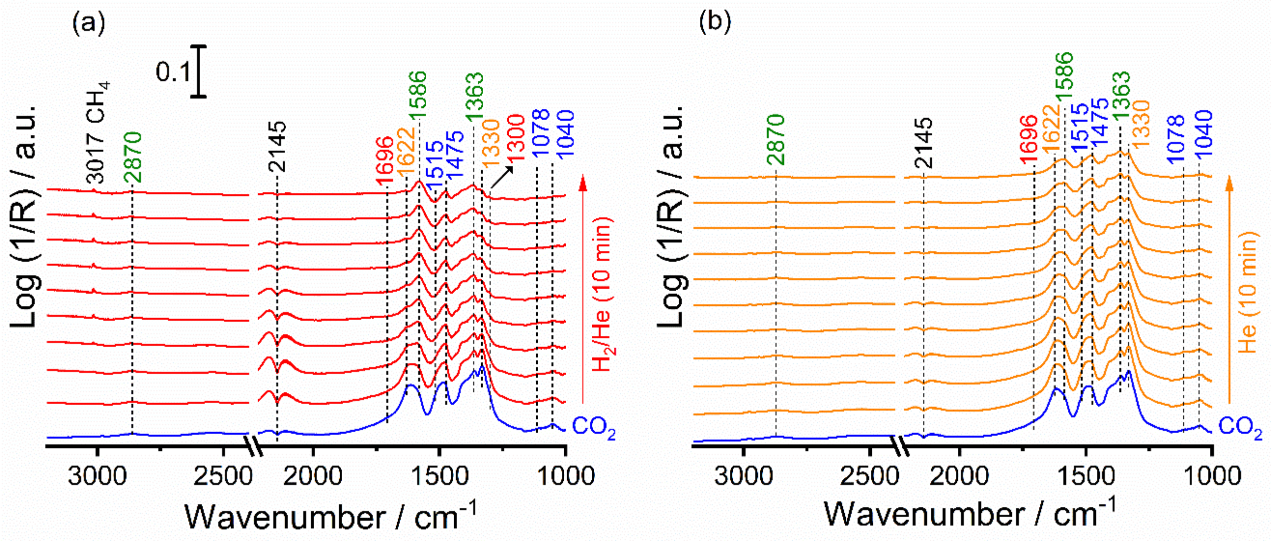

Figure S31. In-situ time-resolved DRIFT spectra obtained over CuZ catalyst when the feed gas was switched between (a) 16.6% CO_2 and 50% H_2 (both in He), and (b) 16.6% CO_2 (in He) and 100% He, at 350 °C under p = 1 bar and a total flow rate of 20 mL·min^{-1}.

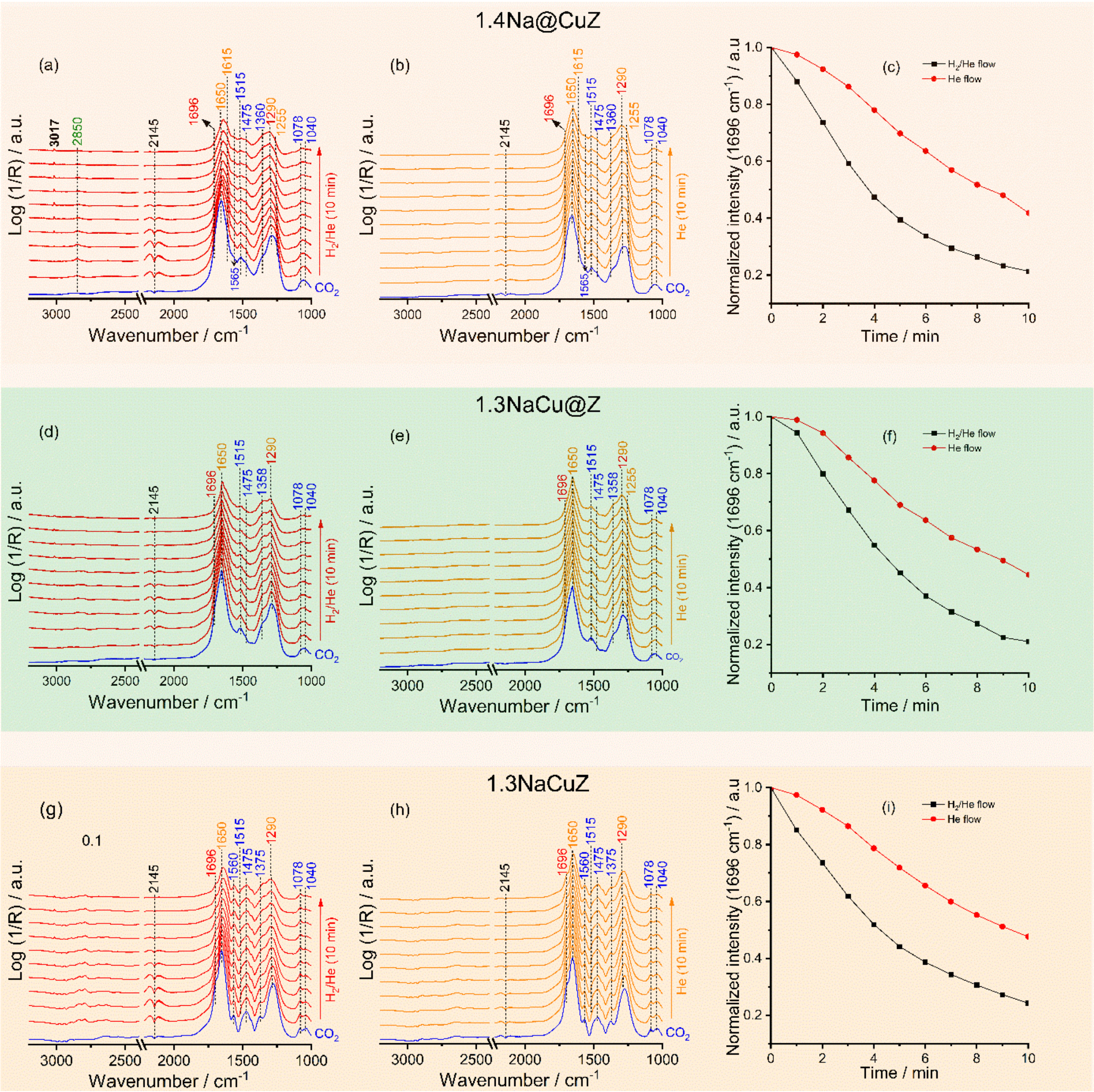

Figure S32. In-situ time-resolved DRIFT spectra obtained over the indicated catalysts when the feed gas was switched between (a, d, g) 16.6% CO_2 and 50% H_2 (both in He), and (b, e, h) 16.6% CO_2 (in He) and 100% He, and (c, f, i) the corresponding normalized peak intensity of carboxylate species (1696 cm^{-1}) under both condition at 350 °C under p = 1 bar and a total flow rate of 20 $mL \cdot min^{-1}$.

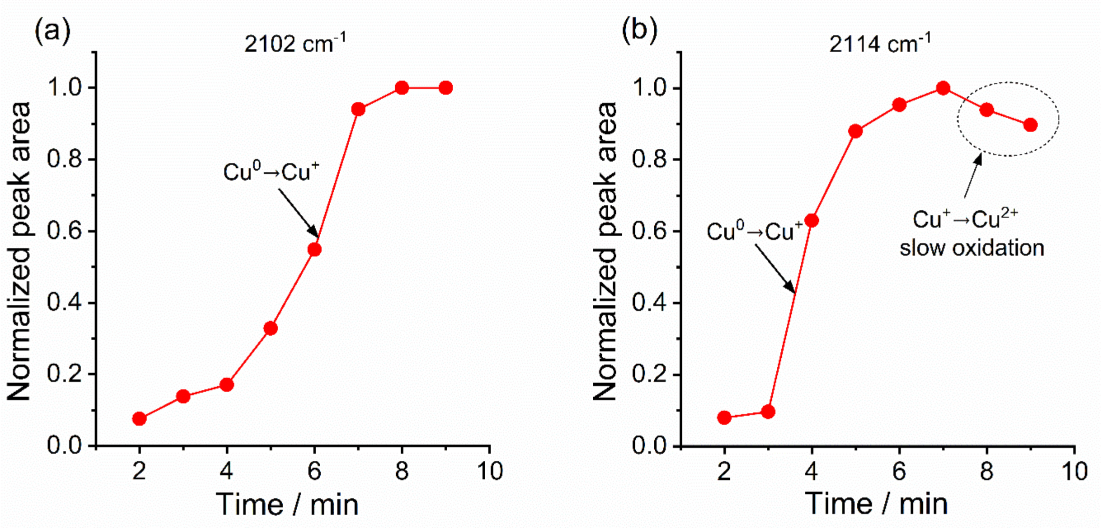

Figure S33. Normalized band areas of the (a) 2102 cm^{-1} band for reduced 1.3NaCuZ and (c) 2114 cm^{-1} band for reduced CuZ during 15% CO_2/He flow at 20 °C for 10 min (corresponding in-situ DRIFTS spectra shown in **Figure 7**).

The normalized band areas showed that, over 1.3NaCuZ, the 2102 cm^{-1} band reached a steady state at 20 °C within 8 min, indicating H_2 is required to reduce Cu^+ back to Cu^0, completing the redox cycle. While, on CuZ, the band at 2114 cm^{-1} slightly declined after reaching its maximum at 7 min, possibly due to further oxidation of Cu^+ to Cu^{2+}, which can not be seen by CO adsorption at 20 °C.

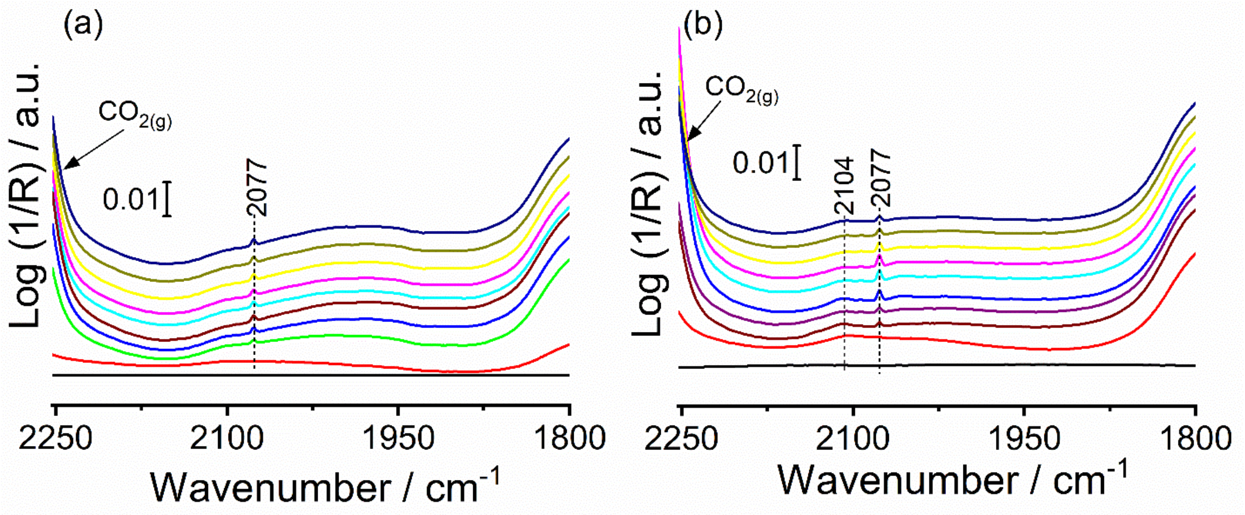

Figure S34. In-situ DRIFT spectra obtained during the flowing of 15%CO_2/He over reduced (a) 1.4Na@CuZ and (b) 1.3NaCu@Z catalysts at 20 °C for 10 min.

Table S1: Elemental composition and surface characteristics of Na-free and Na-modified Cu catalysts supported on ZrO_2.

**Catalysts**	Metal content[a] / wt.%		Phase content[b]/ wt.%		S_{BET}^{c} /$m^2 \cdot g^{-1}$	V_p^{c}/ $cm^3 \cdot g^{-1}$	D_p^{c}/ nm
	Cu	Na	m-ZrO_2	t-ZrO_2			
ZrO_2	---	---	89.1	10.9	72	0.127	4.8
CuZ	0.91	---	79.4	20.6	82	0.142	4.8
0.3Na@CuZ	0.82	0.3	77.1	22.8	75	0.132	4.9
**1.4Na@CuZ**	**0.92**	**1.4**	**76.3**	**23.7**	**69**	**0.126**	**4.9**
1.9Na@CuZ	0.88	1.9	39.9	60.1	62	0.086	3.8
Cu@Z	0.95	---	86.4	13.6	57	0.108	5.1
**1.3NaCu@Z**	**0.90**	**1.3**	**87.1**	**12.9**	**50**	**0.099**	**5.2**
0.4NaCuZ	0.89	0.4	21.6	78.4	95	0.122	3.7
0.9NaCuZ	0.90	0.9	12.9	87.1	98	0.127	3.7
**1.3NaCuZ**	**0.88**	**1.3**	**14.1**	**85.9**	**59**	**0.133**	**5.6**
2NaCuZ	0.88	2	4.5	95.5	57	0.128	6.2

[a] Determined via ICP-OES analysis

[b] determined via XRD patterns

[c] Determined via N_2 adsorption-desorption analysis

Table S2: Surface atomic content (at.%) of the indicated elements determined by ex-situ XPS analysis of the reduced catalysts.

Catalysts	C	O	Zr	Cu	Na
ZrO_2	19.6	51.1	27.5	0.0	0.0
CuZ	17.4	52.0	27.7	1.1	0.0
0.3Na@CuZ	14.5	51.3	26.8	0.9	3.5
**1.4Na@CuZ**	**13.3**	**49.8**	**25.2**	**1.0**	**7.2**
1.9Na@CuZ	11.5	48.4	23.6	0.7	11.0
Cu@Z	19.11	50.59	27.1	1.84	0.0
**1.3NaCu@Z**	**14.39**	**46.74**	**26.2**	**1.52**	**8.24**
0.4NaCuZ	10.0	56.3	30.4	0.5	2.0
0.9NaCuZ	15.2	51.6	26.5	0.5	3.9
**1.3NaCuZ**	**13.1**	**49.6**	**25.9**	**0.9**	**7.9**
2NaCuZ	12.3	49.7	24.0	1.0	10.7

Table S3. Previously reported Cu-based catalysts for the RWGS reaction.

Catalyst	Cu content / wt%	H_2/CO_2	T / °C	GHSV / $mL.g^{-1}.h^{-1}$	CO_2 conversion[b] / %	CO selectivity / %	CO formation rate / $mmole.g_{Cu}.min^{-1}$	Ref.
1.4NaCuZ	0.88	3	300	24000	2.75	100	14.0	This work
			350	24000	10.85		55.0	
			375	24000	18.04		91.5	
			400	48000	16.62		169	
Cu/Al_2O_3	18	3.8	280	10588	7.60	92	0.60	[11]
$Cu\text{-}K/Al_2O_3$	17.1	3.8	280	10588	10.7	99	1.00	[11]
$Cu\text{-}Ba/Al_2O_3$	17.1	3.8	280	10588	16.2	98.1	1.50	[11]
$Cu/\beta\text{-}Mo_2C$	1.3	2	300	300000	5.00	100	286	[12]
1K3CuAlFe	2.4	1	350	30000	14.00	100	65.1	[13]
$Cu/Ce_{0.05}Mg_{0.95}$	9.5	3	350	300000	10.00	100	58.7	[14]
$CuSiO/CuO_x$	15	3	400	60000	4.70	100	3.50	[15]
TiO_2/Cu	15	3	400	60000	0.40	100	0.30	[15]
SiO_2/Cu	15	3	400	60000	0.20	100	0.10	[15]
CuK/C	9	3	260	84545	2.20 (*20*)	100	3.80	[16]
Cu/CeO_2	8.9	3	300	30000	6.00	98	1.20	[17]
Cu/SiO_2	9	3	300	30000	18.00	100	0.30	[17]
$Cu/\gamma\text{-}Al_2O_3$	12	4	400	60000	12.40	100	9.20	[18]
12CuOAl-GD	12	4	400	60000	14.30	100	10.6	[18]
Cu/CeO_2	10	4	350	9000	20.00	100	2.70	[19]
$Cu/CeO_{2-\delta}$	8	4	400	60000	20.10	100	22.4	[20]

Cu/CeO_2-NR	5	5	350	150000	21.00	100	78.1	
Cu/USGO	15	3	300	7440	10.00	100	0.90	[21]
Cu/2DSiO_2-850	15	1	500	18000	22.70	100	10.1	[22]
Cu-ZnO/SBA-15	10	3	340	15000	15.00	100	4.20	[23]
Cu-Fe@Silicate-1 Zeolite	5	3	400	35640	8.00	100	10.6	[24]
8.5Cu 0.3Fe/CeO_2	8.5	1	400	200000	7.00	99	60.7	[25]
1Cs-CuO/CeO_2	20	9	300	30000	13.00	100	1.50	[26]
CuO/CeO_2	20	9	300	30000	20.00	100	2.20	[26]
Cu-Al spinel	35	2	350	60000	21.00	100	8.90	[27]
CuMgAl LDH	9.8[a]	4	300	6000	22.00	100	2.00	[28]
CuIn/ZrO_2	5	4	500	9000	17.00	100	4.60	[19]
$Cu_1Ce_{1.1}$	25.1	4	400	60000	15.46	100	5.50	[29]
Fe-Cu-Cs/Al_2O_3	8	4	400	12504	42.00	100	9.80	[30]
Cu/TiO_{2-x}-600	6	3	300	6000	12.00 (*40*)	70	1.60	[31]
Cu/MnO_2 (Cu:Mn=1)	38.2	5	350	48000	50.90	100	7.90	[32]

[a] The wt% Cu was calculated assuming Cu as metal and Mg and Al in hydroxide form.

[b] The value in parentheses represents the pressure at which the CO_2 conversion was measured.

Table S4. Peak areas of CO adsorption bands observed after CO adsorption at 20 °C for 15 min followed by a He purge for another 15 min.

Catalysts	Absolute area of CO adsorption bands (a.u.)[a]			
	**0.4NaCuZ**	**0.9NaCuZ**	**1.3NaCuZ**	**2NaCuZ**
**H_2-reduced[b]**	2.02	2.03	4.06	0.65
**CO_2-reoxidized[c]**	0.75	0.23	0.93	----

[a] Band areas are compared semi-quantitatively, assuming similar extinction coefficients.

[b] reduced in 50% H_2 at 400°C for 1 h.

c treated with 15% CO_2/He flow at 350°C for 30 min.

References

[1] A. Dandekar, M. A. Vannice, *Journal of Catalysis* **1998**, *178*, 621-639.

[2] O. Dulaurent, X. Courtois, V. Perrichon, D. Bianchi, *The Journal of Physical Chemistry B* **2000**, *104*, 6001-6011.

[3] G. Busca, *Journal of Molecular Catalysis* **1987**, *43*, 225-236.

[4] Y.-Y. Huang, *Journal of the American Chemical Society* **1973**, *95*, 6636-6640.

[5] J. Howard, J. M. Nicol, *Journal of the Chemical Society, Faraday Transactions 1: Physical Chemistry in Condensed Phases* **1989**, *85*, 1233-1244.

[6] J. A. Rodriguez, W. D. Clendening, C. T. Campbell, *The Journal of Physical Chemistry* **1989**, *93*, 5238-5248.

[7] L. H. Dubois, B. R. Zegarski, H. S. Luftman, *Journal of Vacuum Science & Technology A* **1987**, *5*, 455-457.

[8] L. H. Dubois, B. R. Zegarski, H. S. Luftman, *The Journal of Chemical Physics* **1987**, *87*, 1367-1375.

[9] M. J. Kappers, J. T. Miller, D. C. Koningsberger, *The Journal of Physical Chemistry* **1996**, *100*, 3227-3236.

[10] R. A. van Santen, *Journal of the Chemical Society, Faraday Transactions 1: Physical Chemistry in Condensed Phases* **1987**, *83*, 1915-1934.

[11] A. Bansode, B. Tidona, P. R. von Rohr, A. Urakawa, *Catalysis Science & Technology* **2013**, *3*, 767-778.

[12] X. Zhang, X. Zhu, L. Lin, S. Yao, M. Zhang, X. Liu, X. Wang, Y.-W. Li, C. Shi, D. Ma, *ACS Catalysis* **2017**, *7*, 912-918.

[13] M. Gu, S. Dai, R. Qiu, M. E. Ford, C. Cao, I. E. Wachs, M. Zhu, *ACS Catalysis* **2021**, *11*, 12609-12619.

[14] S. Li, X. Liu, J. Ma, F. Xu, Y. Lyu, S. Perathoner, G. Centi, Y. Liu, *ACS Catalysis* **2025**, *15*, 3475-3486.

[15] Y. Yu, R. Jin, J. Easa, W. Lu, M. Yang, X. Liu, Y. Xing, Z. Shi, *Chemical Communications* **2019**, *55*, 4178-4181.

[16] L. Barberis, C. I. Versteeg, J. D. Meeldijk, J. A. Stewart, B. D. Vandegehuchte, P. E. de Jongh, *ACS Catalysis* **2024**, *14*, 9188-9197.

[17] S.-C. Yang, S. H. Pang, T. P. Sulmonetti, W.-N. Su, J.-F. Lee, B.-J. Hwang, C. W. Jones, *ACS Catalysis* **2018**, *8*, 12056-12066.

[18] X. Ai, H. Xie, S. Chen, G. Zhang, B. Xu, G. Zhou, *International Journal of Hydrogen Energy* **2022**, *47*, 14884-14895.

[19] M. Li, T. H. My Pham, Y. Ko, K. Zhao, L. Zhong, W. Luo, A. Züttel, *ACS Sustainable Chemistry & Engineering* **2022**, *10*, 1524-1535.

[20] G. Zhou, F. Xie, L. Deng, G. Zhang, H. Xie, *International Journal of Hydrogen Energy* **2020**, *45*, 11380-11393.

[21] T. T. N. Vu, A. Desgagnés, P. Fongarland, M. C. Iliuta, *International Journal of Hydrogen Energy* **2022**, *47*, 38170-38184.

[22] S. Wang, K. Feng, D. Zhang, D. Yang, M. Xiao, C. Zhang, L. He, B. Yan, G. A. Ozin, W. Sun, *Advanced Science* **2022**, *9*, 2104972.

[23] D. Mao, J. Zhang, H. Zhang, D. Wu, *Catalysis Today* **2022**, *402*, 60-66.

[24] R. Hu, T. Wang, H. Li, Y. Zhu, Y. Wang, F. Wen, E. Xing, Y. Wu, Z. Da, *Catalysts* **2023**, *13*, 1037.

[25] E. Pahija, C. Panaritis, B. Rutherford, M. Couillard, B. Patarachao, J. Shadbahr, F. Bensebaa, G. S. Patience, D. C. Boffito, *Journal of CO_2 Utilization* **2022**, *64*, 102155.

[26] G. Varvoutis, M. Lykaki, E. Papista, S. A. C. Carabineiro, A. C. Psarras, G. E. Marnellos, M. Konsolakis, *Journal of CO_2 Utilization* **2021**, *44*, 101408.

[27] M. Hu, H. Hu, S. Tang, Z. Pan, *Catalysts* **2022**, *12*, 1511.

[28] Y. Chen, H. Hong, J. Cai, Z. Li, *ChemCatChem* **2021**, *13*, 656-663.

[29] G. Zhou, B. Dai, H. Xie, G. Zhang, K. Xiong, X. Zheng, *Journal of* CO_2 *Utilization* **2017**, *21*, 292-301.

[30] L. Pastor-Pérez, F. Baibars, E. Le Sache, H. Arellano-García, S. Gu, T. R. Reina, *Journal of* CO_2 *Utilization* **2017**, *21*, 423-428.

[31] C. Zhang, L. Wang, U. J. Etim, Y. Song, O. M. Gazit, Z. Zhong, *Journal of Catalysis* **2022**, *413*, 284-296.

[32] H. Du, Y. Wang, T. Wan, H. Arandiyan, D. Chu, *ACS Applied Energy Materials* **2018**, *1*, 3035-3041.
